# The E3 Ligases in Cervical Cancer and Endometrial Cancer

**DOI:** 10.3390/cancers14215354

**Published:** 2022-10-30

**Authors:** Fengguang Zhai, Jie Wang, Weili Yang, Meng Ye, Xiaofeng Jin

**Affiliations:** 1 The Affiliated Hospital of Medical School, Ningbo University, Ningbo 315020, China; 2 Department of Biochemistry and Molecular Biology, Zhejiang Key Laboratory of Pathophysiology, Medical School of Ningbo University, Ningbo 315211, China; 3 Department of Gynecology, The Affiliated People’s Hospital of Ningbo University, Ningbo 315040, China

**Keywords:** endometrial carcinoma, cervical cancer, E3 ligase, targeted therapies

## Abstract

**Simple Summary:**

We summarized and cataloged the E3 ligases involved in endometrial carcinoma (EC) and cervical cancer (CC). Finally, an overview of the current drugs that target the ubiquitination process to rescue patients with EC and CC is presented to provide researchers with important research ideas that can be applied to clinical treatment.

**Abstract:**

Endometrial (EC) and cervical (CC) cancers are the most prevalent malignancies of the female reproductive system. There is a global trend towards increasing incidence and mortality, with a decreasing age trend. E3 ligases label substrates with ubiquitin to regulate their activity and stability and are involved in various cellular functions. Studies have confirmed abnormal expression or mutations of E3 ligases in EC and CC, indicating their vital roles in the occurrence and progression of EC and CC. This paper provides an overview of the E3 ligases implicated in EC and CC and discusses their underlying mechanism. In addition, this review provides research advances in the target of ubiquitination processes in EC and CC.

## 1. Introduction

### 1.1. Endometrial Carcinoma

Endometrial carcinoma (EC) is one of the most prevalent malignancies in the female reproductive system. It usually affects fertile or postmenopausal women, especially in developed countries [1]. Disturbingly, the average age of the patients gradually decreased. EC is the third most commonly diagnosed cancer among females aged 20–39 years in the United States, with an annual occurrence rate of approximately 15–25 per 100,000 individuals [2]. Approximately 65,950 EC cases and 12,550 deaths were reported in 2021 [3]. EC’s pathogenesis is influenced by various reasons such as lifestyle, nutrition, obesity, hormonal environment, and age at first pregnancy [4]. Approximately 70% of EC patients are diagnosed with early onset of symptoms and therefore have a good prognosis, with an overall 5-year survival (OS) rate of approximately 77% [5,6]. Conversely, patients with advanced or recurrent EC have a poorer prognosis [5,6]. Depending on its pathogenesis, Bokhman classified EC into two types [7]. One is endometrioid carcinoma with typical symptoms and a good prognosis. The other type is a non-endometrioid carcinoma that is rarer and more invasive, including serous, clear cell, mixed or undifferentiated carcinoma, and carcinosarcoma [8].

In 2013, The Cancer Genome Atlas (TCGA) stratified EC patients for the first time by whole-genome and transcriptome combined microarray, second-generation sequencing, and DNA methylation [9]. Currently, the most common EC histotypes are divided into the following four types: (1) ultra-mutated DNA polymerase εexonuclease tumors (POLE) with a favorable prognosis; (2) microsatellite-unstable tumors or mismatch repair-deficient (MMR) tumor with an intermediate prognosis; (3) microsatellite stability (MSS)or non-specific molecular profile (NSMP) tumor with a moderate prognosis; (4) p53 mutant or high copy-number tumors (serous-like) with poor prognosis [9]. Notably, the subset of ECs diagnosed as high-grade endometrioid carcinoma generally has high copy numbers and mutation profiles, which are more similar to those of plasmacytoid ECs.

### 1.2. Cervical Cancer

Cervical cancer (CC) is the fourth most prevalent cancer worldwide and a major threat to female health. Approximately 150,000 new cases of CC are reported each year, especially in developing countries [10,11]. Although multiple therapeutic approaches, including radiotherapy, chemotherapy, immunotherapy, and targeted therapies, have been developed, the prognosis and survival rate of CC remain poor owing to high metastasis and recurrence [12]. Human papillomavirus (HPV) is the most common causative factor of CC [13]. HPV16 and HPV18 are the two most prominent high-risk viruses. HPV can encode non-structural proteins E6 and E7, which synergistically immortalize infected cervical epithelial cells [14]. In addition, ubiquitination is one of the most critical reversible regulatory mechanisms in biology and is widely involved in HPV protein replication, viral infection, and cell carcinogenesis [15]. For example, HPV E6 and E7 can ubiquitinate p53 and pRb, respectively, and then degrade them, ultimately leading to the malignant transformation of CC.

Fused Toes Homolog (FTS) protein is a variant of the E2 conjugation enzyme, which is closely related to some characteristics of CC [16]. FTS promotes the expression of E6 and E7 in CC cells and then inhibits the expression of p53 and pRb [17]. In addition, the oncogenic features of FTS in CC cells are related to DNA damage repair, phosphorylation of EGFR, activation of Notch1, and spheroid formation, which confer radiation resistance [18,19,20]. FTS is also involved in EGF-induced epithelial-to-mesenchymal transition (EMT) and cell migration in CC cells [21].

### 1.3. Ubiquitination

**Figure 1:**
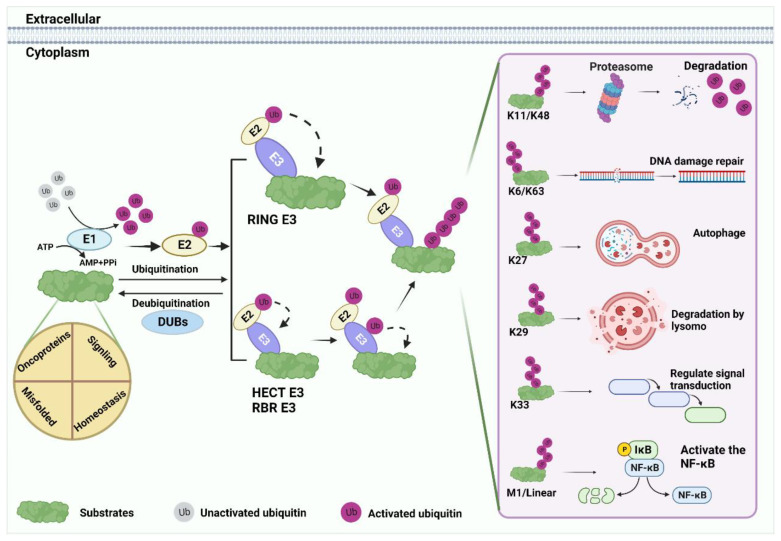
Ubiquitination processes. Substrate ubiquitination is an ordered process. First, under the premise of ATP energy supply, the Ub molecule is activated by E1, and the 76th Gly at the Ub terminus forms a mercaptan lipid bond with E1 cysteine. Subsequently, Ub is shifted to E2 by the E1 intermediate. Finally, E3 ligase transfers Ub directly or indirectly to substrates by recruiting E2. The substrate binds to lysine residues of the Ub polypeptide to form seven poly-Ub chains with different fates: K11 and K48 are commonly associated with substrate degradation. K6, K63, K27, K29, and K33 mediate the regulation of various substrate functions. For example, DNA damage repair, protein autophagy, lysosomal degradation, signal transduction, and so on. M1 and linear Ub chain can positively regulate the nuclear factor kappa-B (NF-κB) signaling.

Ubiquitin (Ub), a small 76 amino acid (aa) protein, contains seven lysine residues that can be covalently labeled with target proteins by either monoubiquitin (mono-Ub) or polyubiquitin (poly-Ub) [13,22,23]. The ubiquitin–proteasome system (UPS) is an important post-translational regulatory mechanism that includes Ub, E1 activating enzyme, E2 conjugation enzyme, E3 ligase, E4 ubiquitin chain assembly factor, 26S proteasome, and deubiquitinase (DUB) [13,14]. Studying different chain types helps to better understand the biological functions of ubiquitination modifications (Figure 1).

Although surgery remains the main treatment for EC and CC, chemotherapy is also an important treatment method. Ubiquitination has been demonstrated to be associated with various cancers, including EC and CC [24]. The UPS regulates the levels and activities of multiple oncoproteins that contribute to the development of EC and CC [25].

## 2. Structure and Function of E3 Ligase Family

More than 600 E3 ligases are known to be associated with mammalian ubiquitination cascade reaction [26,27,28]. They are categorized into four families depending on their structure and function: homologous to the E6-associated protein carboxyl terminus (HECT) domain family, the really interesting new gene (RING) finger family, the RING in-between-RING (RBR) E3 ligases, and U-box E3 ligases [29,30,31,32].

### 2.1. HECT E3 Ligases

The HECT E3 ligase family was first studied [33]. The main distinguishing feature between HECT E3 ligase and other E3 ligases is that its C-terminal contains an E6-related protein homologous domain, which has an active cysteine site that can form an intermediate sulfur-lipid bond with ubiquitin [33,34,35]. The HECT family can be classified into three categories based on its N-terminal structural domains: the HERC family, which has six members; the Nedd4 family, which has nine members; and other HECTs, which have 13 members [36]. The N-terminus of the Nedd4 family contains WW domains that can bind proline-rich and short protein motifs and a C2 domain that can bind Ca^2+^ and phospholipids, which not only promotes protein targeting to the phospholipid membrane but also promotes protein ubiquitination [37,38,39]. The HERC family is comprised of RCC-like domains (RLD) that regulate both GTPase Ran or interface with chromatin [40,41,42]. In addition, there are other HECT ligases, such as E6-associated protein (E6AP) and HUWEI [43,44]. In summary, among all mammalian E3 ligases, the HECT domain determines ubiquitination specificity. It plays essential roles in several signaling pathways associated with the transport of many receptors, regulation of immune responses, and cell proliferation [36].

### 2.2. Other E3 Ligases

The RING finger family has the highest number of E3 ligases, characterized by its RING domain. Its main members include cullin-ring ligases (CRLs) and the anaphase-promoting complex (APC) [26,45,46]. CULs, RINGs, adaptor proteins, and substrate recognition adaptors are the four main components of CRLs [47]. At present, only two RING structural proteins, RBX1/ROC1 and RBX2/ROC2/SAG, have been found to participate in CRL formation. The substrates of the RING finger family are associated with various cellular processes, such as cell proliferation, metabolism, apoptosis, differentiation, and DNA repair [48]. In addition, the RING finger family can regulate its function and activity by interacting with different small molecules, including autoubiquitination, neddylation, and phosphorylation [28].

RBR E3 ligase is a unique RING-HECT heterozygous E3 ligase family, which differs from the RING and HECT families [49]. The RBR E3 ligase is characterized by its conserved RBR domain, including the RING1, central intermediate ring (IBR), and RING2 domains [50]. Although RBR E3 ligases function in a manner similar to that of HECT E3 ligases, RBR ligases tend to ubiquitinate substrates via linear Ub chains, which is a unique mechanism [28].

U-box E3 ligases are poorly studied in mammals [51]. The C-terminus of the U-box E3 ligase has a conserved U-box structural domain, which consists of approximately 70 aa residues in yeast and humans [52]. The process of ubiquitination catalyzed by U-box E3 ligases is defined as the direct transfer of Ub from E2 to target proteins through interaction with E2 through the U-box domain [53]. U-box E3 ligases are essential for the post-translational quality control of proteins in cells. They often promote the degradation of misfolded and unfolded proteins [54]. For example, recent studies have shown that CHIP can inhibit prostate cancer (PCa) by degrading androgen receptor (AR) [55,56].

## 3. E3 Ligases in Signaling Pathways Associated with EC and CC

**Figure 2:**
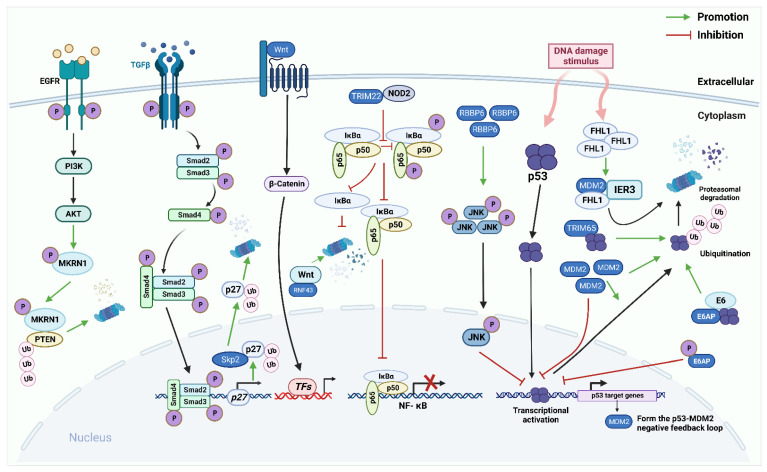
The role of E3 ligases in the p53 pathway, the Nuclear Factor Kappa-B (NF-κB) pathway, the EGFR pathway, the Wnt/β-catenin pathway, the TGF-β/Smad pathway in endometrial carcinoma (EC) and cervical cancer (CC). The p53 signaling pathway: E3 ligases murine double minute 2(MDM2), E6AP, and TRIM65 directly bind to p53 and promote ubiquitination degradation of p53. p53 protein, in turn, induces MDM2 to regulate itself through self-ubiquitination. Furthermore, RBBP6 promotes the phosphorylation of c-Jun NH2-terminal kinase (JNK) to regulate the expression of p53. In CC cells, FHL2 binds to IER3 and MDM2 to form a ternary complex that allows for efficient MDM2-mediated degradation of IER3 by enhancing the association between MDM2 and IER3. NF-κB pathway: E3 ligase TRIM22 directly binds to nucleotide-binding oligomerization domain-containing protein 2 (NOD2). Reducing the phosphorylation of p65 and IκBα reduces NF-κB signal transduction. Epidermal growth factor receptor (EGFR) pathway: makorin RING finger protein 1 (MKRN1) can be phosphorylated by the EGFR/PI3K/AKT pathway to continuously degrade PTEN protein in a K48-dependent manner. Wnt/β-catenin pathway: RNF43 directly interacts with Wnt and promotes its degradation, thereby inhibiting continuous activation of the Wnt/β-catenin pathway. TGF-β/Smad pathway: Activated TGF-β ligand interacts with two transmembrane receptors TβRI and TβRII, to form a tetrameric complex. Constitutively activated TβRII phosphorylates and activates TβRI, which directly phosphorylates downstream transcription factors Smad 2 and Smad 3 through its serine/threonine kinase activity. Activated Smad2 or Smad3 can bind to Smad4, migrate to the nucleus, and bind to different TFs to achieve excessive activator or suppressor activity. SCF^Skp2/Cks1^ directly interacts with p27 and ubiquitinate it.

Increasing evidence indicates that critical protein ubiquitination in various signaling pathways plays a crucial role in the development of EC and CC. This manuscript summarizes the relationship between ubiquitination and some important EC and CC-related signaling pathways, such as the p53, the Nuclear Factor Kappa-B (NF-κB), the epidermal growth factor receptor (EGFR), the Wnt/β-catenin, and TGF-β/Smad pathways (Figure 2, Table 1).

### 3.1. p53 Pathway

p53 dysfunction in malignant tumors is mainly due to the inactivation of the p53 protein by mutations in binding proteins or TP53. p53, a tumor suppressor protein, is a potent transcription factor that promotes cell cycle arrest, senescence, and apoptosis by regulating more than 100 target genes in response to various stimuli [75]. Although p53 regulates the expression of several tumor suppressor proteins, it is usually retained in an “off” state because of its rapid turnover [76]. Monoubiquitination of the C-terminal domain of p53 acts not only as a scaffold for polyubiquitination but also as a signal for the nuclear export of p53, which is the foremost step in limiting p53 function [77,78,79]. In the cytoplasm, multiple E3 ligases lead to the ubiquitination of p53 and the degradation of the 26S proteasome [80]. Thus, the critical role of E3 ligases in the p53 signaling pathway associated with EC and CC is comprehensively summarized.

#### 3.1.1. MDM2

Murine double minute 2 (MDM2) is a well-known oncogenic protein, which is composed of an N-terminal p53-binding domain, nuclear localization signal (NLS), nuclear export signal (NES), acidic domain, zinc finger domain, and C-terminal RING finger domain [81]. The RING finger domain, which is located in the C-terminal, is the main functional domain [82]. MDM2 acts as a cancer-promotor in EC and CC by regulating the stability of p53 [57,58]. MDM2 not only plays a critical role in recognizing the N-terminal trans-activating domain (TAD) of p53 but also acts as a p53 transcriptional activation repressor [83]. Furthermore, MDM2 phosphorylation appears to be a key step in mediating p53 degradation and preventing its ubiquitination [84,85]. The mechanism associated with MDM2 and p53 degradation is best shown in mouse models, in which the inactivation of p53 completely rescues the embryonic mortality of MDM2 when its function is lost [86]. The prototype small molecule CP-31398, a synthetic styrene quinazoline, can inhibit EC proliferation and migration by down-regulating MDM2 expression and stabilizing p53 activity [87]. Previous studies have shown that CP-31398 blocks the migration and invasion of hepatocellular carcinoma (HCC), colorectal cancer, and PCa caused by p53 deficiency both in vitro and in vivo [88,89,90]. Although there are few studies on the relevant mechanisms of MDM2 in promoting cancer in EC and CC, the existence of substrates other than p53 or molecules that indirectly interact with p53 requires further exploration.

#### 3.1.2. E6AP

Almost all cervical precancerous and malignant squamous cell lesions are associated with HPV infection. High-risk HPV-expressed E6 oncoprotein can recruit E6AP and subsequently induce 26S proteasome degradation of p53, which is a key factor in CC cell transformation [59]. E6AP belongs to the HECT family, with HECT domains involved in binding substrates and distinct N-termini that mediate substrate specificity [91]. The E6/E6-AP complex regulates various HPV-induced oncogenic proteins through proteasome [92]. These oncogenic proteins include E6 targeting protein 1(E6TP1), Bcl-2 antagonist killer (Bak), and cellular myelocytomatosis oncogene(c-Myc), which will continue to be extended as more research is conducted on the role of E6AP [14]. p53 mutations frequently occur in cancer cells and are concentrated in the DNA-binding domain [93]. A study by Li et al. emphasized that the tumor suppressor function of wild-type p53 could be established using a p53 mutant [59]. A recent study showed that E6 phosphorylation inhibits the transcriptional activity of p53 [94]. The PSD-95/Dlg/ZO-1 binding motif (PBM) of the C-terminal of E6 contains a well-characterized phosphate receptor site that, in the absence of E6AP, leads to a significant increase in p53 levels and phosphorylated E6 (pE6) levels, which in turn inhibits p53 transcriptional activity [59]. Thus, a negative feedback loop is formed.

#### 3.1.3. TRIM65

TRIM65, which belongs to the RING E3 ligase family, is a 517 aa length protein [49]. TRIM65 is widely associated with white matter lesions, innate immunity, tumors, and other diseases [49]. The study by Wang et al. reported that TRIM65 is dramatically overexpressed in human CC tissues [13]. In vitro experiments have demonstrated that TRIM65 can interact with p53 and promote p53 ubiquitination, thereby inhibiting the apoptosis of CC cells. In addition, the overexpression of TRIM65 can promote mTOR phosphorylation, and this process will block the degradation of the pathogen and decrease autophagy-related apoptosis.

#### 3.1.4. RBBP6

Retinoblastoma-binding protein 6 (RBBP6) belongs to the RING E3 ligase family and is closely related to apoptosis, cell cycle regulation, and tumorigenesis [95,96]. Three subtypes can be encoded by the *RBBP6* gene: Rb, the RING domain, zinc, and p53 binding domains comprise subtype1 and 2, which serve as E3 ligases; subtype 3 contains a domain with no name (DWNN) domain, which possesses the ability of ubiquitin-like modification [97,98]. RBBP6 regulates p53 protein levels and activity through three pathways. First, RBBP6 interacts directly with p53 to regulate gene transcription [95]. Second, RBBP6 promotes apoptosis through ubiquitination and degradation of Y-box binding protein 1 (YB-1), which can serve as a transcription factor and associate with the suppression of p53 [99]. Third, RBBP6 interacts with the E3 ligase MDM2 and enhances its ability to mediate p53 ubiquitination and proteasome degradation [100]. RBBP6 was overexpression in CC tissues. In vitro experiments showed that when the expression of RBBP6 was upregulated, the c-Jun NH2-terminal kinase (JNK) phosphorylation increased, and the transcription of p53 was inhibited, leading to a significant increase in the viability, migration, and proliferation in CC cells [60].

### 3.2. NF-κB Pathway

NF-κB belongs to the transcription factor family, which is expressed in almost all animal cell types and activated by various signaling proteins. It is a major pro-inflammatory signaling pathway required for the rapid activation of immune cells [101,102]. The NF-κB pathway is associated with carcinogenesis, inhibition of apoptosis, and promotion of resistance to anticancer drugs [103]. Various stimuli can activate the classic NF-κB pathway, such as TNFα, LPS, IL-1β, IL-1R, TLR, TNFR, and antigen receptors, which in turn promote the activation of IκB kinase β (IKKβ). IKKβ activation can promote IκBα phosphorylation, which mediates IκBα ubiquitination. The release and translocation of NF-κB dimers promote nuclear drive target genes transcription [104]. Current studies have found that NF-κB induced inflammation promotes the development of EC. In addition, NF-κB signaling pathways are regulated by various E3 ligases, such as CUL3^SPOP^, TRIM22, and TRIM25.

#### 3.2.1. CUL3^SPOP^

SPOP is a substrate adaptor of the complex of CUL3-RBX1 E3 ligase [105]. Substrates that can be ubiquitinated in human cells include death dome-associated protein (DAXX), phosphatase (PUC), steroid receptor coactivator(SRC-3) and transcriptional regulator (CI/GLI), estrogen receptor-α (ERα), and BRD2, BRD3, and BRD4 proteins (BETs) [25,106]. SPOP is mutated in 8.7% of patients with EC, based on the data available in the cBioPortal portal (https://www.cbioportal.org (accessed on 23 September 2022)). These mutations occur mainly outside the cleavage on the substrate-binding surface in the MATH region [107]. ERα, encoded by the *ESR1* gene, is highly overexpressed in hormone-responsive cancers, such as ovarian, endometrial, and breast cancers [108]. A previous study showed that CUL3^SPOP^ interacts with ERα and triggers its ubiquitination and degradation, thus suppressing the occurrence and progression of EC. Then, this process inhibits the cancer-promoting effects of ERα in EC [105]. However, EC-related CUL3^SPOP^ mutants are flawed in the ubiquitination of ERα [105]. Another study showed that CUL3^SPOP^ could ubiquitinate and degrade BETs proteins, particularly BRD3, in PCa and EC [106]. Controversially, PCa-associated CUL3^SPOP^ mutations inhibit BET protein degradation, whereas EC-associated CUL3^SPOP^ mutations potentiate BET protein degradation [106,109]. Currently, the functional gain mechanism and precise structural basis of EC-related CUL3^SPOP^ enhancing BET protein degradation need to be further elucidated. Death-associated protein kinase-related apoptosis-inducing kinase 1 (DRAK1) can act as a potential positive regulator of apoptosis. DRAK1 depletion enhances TRAF6 protein expression, which ultimately activates NF-κB signaling and promotes oncogenesis in CC cells [61]. Pang et al. demonstrated that the down-regulation of DRAK1 expression is usually accompanied by paclitaxel resistance in CC cells. Furthermore, increasing the protein expression level of DRAK1 could make CC cells more sensitive to carboplatin and paclitaxel [110]. Mechanistically, CUL3^SPOP^ mediates DRAK1 proteasome degradation through K48-linked polyubiquitin, leading to increased TRAF6 levels. TRAF6 then promotes CC progression by activating the NF-κB signal pathway.

#### 3.2.2. TRIM22

Tripartite motif-containing 22 (TRIM22) contains three main domains: coiled-coil, B-box, and a RING finger [62]. TRIM22 also associates with various biological processes in transcription regulation [111] TRIM22 expression is downregulated in EC tissues, which is associated with high malignancy and poor prognosis [112,113,114]. Zhang et al. confirmed that TRIM22 could induce and promote nucleotide-binding oligomerization-domain-containing protein 2 (NOD2) expression, reduce the phosphorylation of P65 and IκBα, resulting in reducing NF-κB signal transduction, and ultimately inhibit EC development and progression [62]. NOD2 belongs to the NOD-like receptors (NLRs) family that is stimulated by TLR to induce IRF4, which subsequently inhibits NF-κB activation through interactions with MyD88 or TRAF6, enabling NOD2 to inhibit NF-κB signaling [115,116]. *TRIM22* can be used as a progesterone target gene to increase EC patients’ prognosis [117]. In conclusion, TRIM22 is a significant predictor for prognostic and also is a potential therapeutic target for patients with EC. Comprehensive and in-depth studies on the potential mechanism of TRIM22 downregulation in patients with EC are urgently required.

#### 3.2.3. TRIM25

TRIM25 is structurally similar to TRIM22 and belongs to the TRIM protein superfamily [118]. TRIM25 has been confirmed to contribute to the aggressiveness of various cancers. For example, in breast cancer cells, TRIM25 degrades the cell cycle checkpoint protein 14-3-3σ via UPS. Thereby, this process will promote the formation of xenograft tumors in mice [119]. TRIM25, an interferon (IFN) responsive gene, modulates the NF-κB pathway [120,121]. Sato et al. demonstrated that TRIM25 degrades 14-3-3σ protein through ubiquitination, thereby enhancing NF-κB signaling and the related factors which can promote the development of EC [63]. The expression level of TRIM25 is reduced in EC tumors and positively related to the ERα expression, suggesting that the function of TRIM25 on EC may be related to ERα signal transduction [122,123]. However, in vitro TRIM25 down-regulation reduced EC cell migration in ERα+ and ERα- cell lines, regardless of estrogen treatment. Therefore, the natural role of TRIM25 in EC requires further investigation.

### 3.3. EGFR Pathway

Epidermal growth factor receptor (EGFR) is a member of the ErbB family and an important signaling pathway activating receptor on the cell membrane surface. EGFR dimerization activates PI3K/AKT and other signaling pathways through the phosphorylation cascade to play biological functions. Phosphatase and tensin homolog (PTEN), as a tumor suppressor, is a potent inhibitor of the PI3K/AKT signaling pathway because PTEN promotes the dephosphorylation of phosphatidylinositol-3, 4, 5-triphosphate (PIP3) to phosphatidylinositol-4, 5-diphosphate (PIP2) [124]. The crosstalk between the phosphorylation cascade and ubiquitination affects various cellular physiological processes [125]. For example, Li et al. found that the EGFR/PI3K/AKT/makorin RING finger protein1 (MKRN1)/PTEN axis plays an important role in the occurrence and development of CC [64]. MKRN1 is an E3 ubiquitin ligase that can be more stable via being phosphorylated by pAKT at S109 [64], while phosphorylated MKRN1 persistently induces polyubiquitination and degradation of the K48-linked PTEN [64].

### 3.4. Wnt/β-Catenin Pathway

The Wnt signaling pathway plays a role in a wide range of animal cells and is a highly conserved signaling pathway in species genetics. Persistent abnormal Wnt activation or abnormal accumulation of β-catenin is a key process in tumorigenesis [126,127]. It has been demonstrated that E3 ligase promotes EC development by labeling critical molecules of this pathway, such as RNF43 [128,129].

RNF43 is a type I integral membrane protein containing a RING finger domain with the vast majority of its aa motifs located in the intracellular region [130]. RNF43 is a negative regulator of the Wnt signal. It and the related side family homolog ZNRF3 can ubiquitinate Wnt receptors to function [128,129]. By querying the COSMIC cancer database, we find that the most common RNF43 mutations are nonsense or shift mutations. In addition, the most common truncating mutation is G659Vfs*41. The frequency in EC is about 5–8% [65]. Treatment for patients with RNF43 mutated EC mainly focuses on sustained inhibition of Wnt signaling by inhibiting the production of Wnt ligands. However, no study has demonstrated the feasibility of such a scheme.

### 3.5. TGF-β/Smad Pathway

The transforming growth factor-β (TGF-β) signaling pathway plays an important physiological function in maintaining normal cellular physiology and promoting embryonic maturation and development, among other processes. Abnormal signaling of TGF-β, a potent endogenous proliferation inhibitor, occurs in the development of many human type I EC [131]. Activated TGF-β ligand interacts with TβRI and TβRII, which can form a tetrameric complex. Constitutively activated TβRII phosphorylates and activates TβRI, which directly phosphorylates downstream transcription factors Smad2 and Smad3 through its kinase activity [132]. Active Smad2 or Smad3 can bind to Smad4, then migrate to the nucleus. They also can bind to different transcription factors (TFs) to achieve excessive activator or suppressor activity [133]. In general, the antitumor effects can be achieved by inactivating essential components of this signaling pathway, such as p27kip1 (p27) [134].

APC and the SCF^Skp2/Cks1^ complex are two major E3 ligases that can regulate cell cycle progression [135,136]. In normal endometrial epithelial cells, TGF-β promotes APC binding to Cdh1, forming an E3 ligase complex capable of ubiquitination of SCF^Skp2/Cks1^, thereby reducing the degradation of nuclear p27, which then inhibits cyclin-dependent kinase 2(Cdk2) to prevent EC cell growth [137]. Sustained ubiquitination of p27 by SCF^Skp2/Cks1^ is an early causative event of type I EC. Furthermore, estrogen promotes phosphorylation of the Thr187 site of p27 in a MAPK/erk2-dependent manner, which is required for SCF^Skp2/Cks1^ to recognize and ubiquitinate p27 [66]. In contrast, progesterone inhibits estrogen-induced endometrial proliferation during the menstrual secretory phase [138]. Therefore, progesterone has been identified as a treatment drug for type I EC.

## 4. Other E3 Ligases

### 4.1. SCF^FBXO2^ and SCF^FBXW7^

The F-box protein is an adaptor of the Skp1-CUL1-F-box ubiquitin ligase complex, which is characterized by approximately 40 aa motifs [139]. It is involved in cell growth, development and differentiation, signaling responses, and cell survival and death by degrading cancer-related regulatory proteins [140]. The F-box protein dysregulation can lead to sleep loss, diabetes, Parkinson’s disease, and infectious diseases [141,142,143]. Currently, it can be divided into three families according to the differences in recognizable domains: the F-box and WD40 domain (FBXW) family contains the WD-40 domain, the F-box and Leu-rich repeat (FBXL) family is rich in leucine repeats, and the remaining family belongs to the F-box only (FBXO) family. Notably, F-box proteins are associated with various biological pathways, with the most well-defined biological roles and properties of EC being FBXO2 and FBXW7.

SCF^FBXO2^ is predominantly present in neurons and mediates related glycoproteins ubiquitination, thereby regulating their associated functions and safeguarding the normal function of neuronal cells [144,145]. Furthermore, the Fox-associated domain (FBA) is crucial for its glycoprotein-recognizing activity. It can bind to the N-linked high-mannosylglycan fraction marked by glycoproteins [145]. Recently, the roles of SCF^FBXO2^ have been experimentally confirmed in diverse cancers. For instance, SCF^FBXO2^ regulates the EMT in gastric cancer. Of concern, in colorectal cancer, SCF^FBXO2^ has been used to predict poor prognosis [146,147]. Che et al. demonstrated that overexpression of FBXO2 patients with EC is associated with various poor clinical indicators. SCF^FBXO2^ can interact with fibrillin, which is considered an essential tumor suppressor through the FBA domain and mediates its ubiquitination and degradation [67]. In addition, their data suggest that the abnormal expression of SCF^FBXO2^ is associated with the cell cycle and autophagy signaling pathways.

SCF^FBXW7^, also known as SCF^FBXW7^ and Cdc4, acts as a tumor suppressor and is associated with at least 35 tumor types [14]. FBXW7 can be divided into three main isoforms according to their N-termini difference. FBXW7α localizes to the nucleoplasm, which is the most abundant isoform. FBXW7β localizes to the cytoplasm. FBXW7γ isoforms localize to the nucleolus [148,149]. SCF^FBXW7^ maintains normal cellular biological functions in normal cells by degrading several carcinogenic regulators that target proliferation, growth, and apoptosis, including Notch 1, cyclin E (CCNE1), c-Myc, Aurora kinase A, and Jun [150]. Mutations in *FBXW7* is approximately 6% of all kinds of cancers, the most common of which is T-cell acute lymphoblastic leukemia (T-ALL; 31%) [151,152]. In addition, a novel nonsense mutation has been confirmed to be associated with colorectal cancer. In EC, missense mutations are the predominant form of *FBXW7* mutations (about 16%) [153].

Even though the frequency of *FBXW7* mutations in EC is high, the individual context and molecular consequences of such mutations remain poorly understood. Kuhn et al. proved that the existence of FBXW7 mutation and CCNE1 amplification raised the level of CCNE1 protein, thereby promoting cell proliferation [68]. Therefore, future work should explore more molecular biological effects of EC-associated FBXW7 mutations and determine whether FBXW7 mutations can be used as precision therapeutic targets to develop targeted therapeutic drugs. This work may be critical for improving patient care with clinically aggressive EC.

### 4.2. APC^Cdc20^

Evidence has emerged that APC is the predominant driver in the control of cell cycle progression [154]. The APC core is interlinked with the activator cell division cycle 20 homolog (Cdc20), allowing it to form a complex capable of biological function [155]. Emerging evidence suggests that Cdc20 recruits its substrates through different motifs, such as the destruction box (D-box), TEK, and the newly identified ABBA [156,157,158,159]. APC^Cdc20^ is known to exhibit carcinogenic effects in various cancers, including breast, hepatocellular, lung, bladder, and endometrial [160]. The gene encoding the 17-β-estradiol (E2) oncoprotein is often inactivated in CC cells infected with high-risk HPV-18. In addition, E2 can inhibit two viral oncogenes’ expression, E6 and E7, which are the main steps in the HPV-induced transformation of CC cells [161]. Although E2 can interact with Cdc20 and Cdh1, two co-activators of APC, E2 is not ubiquitinated by APC. Instead, E2 can inhibit APC function, allowing substrates accumulation, such as SCF^Skp2/Cks1^. In the late G1 phase, SCF^Skp2/Cks1^ directly interacts with E2 and mediates proteasomal degradation [162]. Simultaneously, it also promotes E6 and E7 expression and induces HPV-infected cells’ early entry into the S phase. Yu et al. showed that the HPV16 E7 oncoprotein can highly express and inhibit EMI1 degradation in mitotic cells by promoting the APC inhibitor EMI1. Overexpression of EMI1 suppresses substrate degradation and prophase delay of mitosis [163]. Long noncoding RNA (lncRNAs) plays an essential role in cells by influencing protein ubiquitination [65,164,165,166,167]. Studies report that lncRNA-ZXF1 has been shown to be an important regulator of EC development [69]. In normal endometrial cells, lncRNA-ZXF1 can interact with P21 to stabilize the protein level of P21 and avoid its degradation by APC^Cdc20^ [69]. Therefore, lncRNA-ZXF1 may be an important prognostic indicator for precision therapy because of its tumor suppressor effect in EC.

### 4.3. HUWE1

HUWE1, an E3 ligase with a catalytic HECT domain and a substrate-binding loop. The number of substrates that can be ubiquitinated by HUWEI1 has exceeded 40. It is closely related to cell stress responses, cell statue, signal transduction, inflammasome activation, and other cellular processes [168]. Comprehensive molecular studies have shown that HUWE1 can target various substrates, including c-Myc [169]. In CC, the expression level and activity of HUWE1 are significantly down-regulated, which promotes c-Myc expression and cell propagation [70]. Specific inhibitors of c-Myc against various human tumors are in stage I/II clinical trials. However, because the discovery of c-Myc inhibitors is difficult, BET inhibitors are currently used as an alternative strategy in preclinical studies. For example, the BET inhibitor GS-626510 could effectively inhibit xenograft tumor growth in vivo [70].

### 4.4. Cullin2^E7^

High-risk HPV E7 is closely related to the development of CC. Typically, E7 exerts its oncogenic potential by inactivating the normal function of tumor oncogenic factors [170]. Other properties of E7 suggest its involvement in virus-induced cell transformation. Rb protein plays an important role in the development of CC, and it can be degraded by E7-mediated ubiquitination. Similar to E6-mediated p53 ubiquitination degradation, E7 recruits E3 ligase to target Rb leading to its ubiquitination and degradation in CC [171]. At present, Cullin2 is the only E3 ligase that can bind to E7 to form a complex, but there is no evidence in vitro [172]. Notably, Wang et al. found that a novel Rb E3 ligase (NRBE3) promotes Rb degradation via K48-dependent ubiquitination in vivo and in vitro [173]. Whether NRBE3 can bind to HPV E7 to induce Rb degradation in CC is worthy of further exploration.

### 4.5. MDM2

Immediate early response 3 (IER3) can rapidly induce apoptosis of CC cells in various ways [174,175,176,177]. In CC cells, MDM2 directly binds with IER3 and mediates its K60 polyubiquitination [71]. Four and a half LIM domain-containing protein 2 (FHL2) is a multifunctional protein associated with various cellular biological processes, including gene manifestation regulation, cell statue, propagation, differentiation, adhesion, and migration [178,179,180]. Due to its structure, FHL2 acts as a binding site that interacts with different proteins and forms complexes [179,181]. In CC cells, FHL2 binds to IER3 and MDM2 to form a ternary complex that allows the efficient MDM2-induced degradation of IER3 by enhancing the association between MDM2 and IER3 [71].

### 4.6. RNF114 and Smurf1

RING finger 114 (RNF114) and Smurf1 are members of the E3 ligase family. Studies have demonstrated that RNF114 can positively regulate the activity of poly (ADP-ribose) polymerase 10 (PARP10) through K27-linked polyubiquitination in CC [72]. Zhao et al. identified PARP10 as a tumor metastasis suppressor and demonstrated that PARP10 interacts with Aurora A and then inhibits its kinase activity, thereby regulating Aurora A downstream signaling to inhibit the EMT process in tumor cells and tumor metastasis [72,182]. Interestingly, the G888W mutant of PARP10 blocks its interaction with RNF114 and abolishes the enzymatic activity of PARP10 [183]. Many studies have confirmed that FOXA2 is crucial for inhibiting tumor metastasis by promoting EMT [184,185]. Staphylococcal nuclease domain-containing protein 1 (SND1) is highly expressed in CC tissues and is positively correlated with a shorter survival time [73]. Cell experiments confirmed that SND1 promotes Smurf1 expression in CC cells. Smurf1 interacts with FOXA2 and induces its ubiquitination and proteasome degradation, an essential mechanism by which SND1 promotes metastasis in CC cells [73].

### 4.7. NEDD4L

NEDD4L not only regulates the function of epithelial sodium channels to achieve cell stability and homeostasis but also prevents cancer development by inhibiting the transforming growth factor-β(TGF-β) signaling pathway [186]. TGF-β is a multifunctional polypeptide growth factor that plays a tumor suppressor role in normal and precancerous cells but plays a cancer-promoting role in cancers [187]. Yilmaz et al. were the first to study NEDD4L expression in EC, and their findings showed that NEDD4L expression levels are reduced in EC patients compared with benign endometrial lesions [74]. This finding implied that NEDD4L may play an essential role in the occurrence and development of EC. Therefore, it is necessary to explore new substrates or pathways related to NEDD4L to provide new clues for inhibiting the occurrence and development of EC.

## 5. Mutations in E3 Ligases in EC and CC

Mutations are also important causes of cancer. However, E3 ligase mutations in EC and CC have not been systematically studied and summarized. We searched and summarized the status of E3 ligases leading to EC and CC using the cBioPortal database. However, the specific effects of these mutated sites on E3 ligase, such as activation or inactivation, have not been confirmed (Table 2 and Table 3).

Considering these mutation sites and mutation types, we found that these E3 ligases are dominated by missense mutations in EC and CC and are mainly found in EC. The discovery of new mechanisms of E3 ligase mutation and the development of EC and CC can provide new therapeutic targets for subsequent treatment. Therefore, other E3 ligases can be similarly explored and further explored in their functions to help find better therapeutic targets.

## 6. Treatment

Increasing evidence suggests that E3 ligases are related to the development of EC and CC, two highly lethal gynecological malignancies, at multiple levels. Although this review summarizes and catalogs the E3 ligases involved in EC and CC, the genetic alterations of these components in human diseases have not been fully evaluated. Therefore, identifying therapeutic targets for new drugs is very important for the development of therapeutic strategies.

### 6.1. Targeting Proteasomes for EC and CC Therapy

Bortezomib was the first selective proteasome inhibitor approved for clinical, which has led to the development of many other proteasome inhibitors and has opened up a new approach for treating human malignant tumors by regulating the UPS [188,189]. A previous study showed that bortezomib inhibited EC cell proliferation and proteasome activity and induced G2/M phase arrest and apoptosis [190]. Interestingly, sorafenib can also inhibit EC development [191]. However, sorafenib enhanced the activity of UPS through the AKT/GSK3β and ERK pathways and promoted the degradation of the anti-apoptotic protein Mcl-1. As potential anticancer drugs, HIV protease inhibitors (HIV-PIs), such as lopinavir and indinavir, have been shown to selectively inhibit the 26S proteasome in CC [192,193]. Mechanistically, these protease inhibitors damage the cellular proteasome by targeting chymotrypsin activity at the protein breakdown rate, ultimately preventing the degradation of p53 and increasing the levels of tumor suppressor proteins [194,195,196,197]. Although drugs targeting the 26S proteasome remain at the cellular level for the treatment of EC and CC, they also provide an experimental basis for subsequent clinical applications. Similarly, the development of small-molecule inhibitors (SMIs) that can specifically target E3 ligases, thereby reducing the ubiquitination of its substrate, is currently the most promising therapeutic approach. Notably, owing to the specificity and diversity of E3 ligases, inhibitors targeting E3 ligases have lower cytotoxicity and better therapeutic efficacy than proteasome inhibitors.

### 6.2. Targeting E3 Ligases for EC and CC Therapy

#### 6.2.1. Patient-Derived Tumor Xenograft (Pdx)

E3 ligases have received increasing attention as therapeutic targets in EC and CC cells. However, SMIs of these identified E3 ligases involved in EC and CC have been mainly focused on fundamental research and have not been validated for clinical trials and applications. The currently developed SMIs capable of targeting E3 ligases have shown significant antitumor activity in mouse tumor xenograft models, such as XI-001. The pseudourea derivative XI-001 compounds identified as p53 activators by high-throughput screening were used to kill cancer cells but did not affect normal cells [198,199]. Zhang et al. demonstrated that XI-001 could inhibit the growth of xenograft tumors in HeLa tumor-bearing mice and enhance the cytotoxic activity of cisplatin in vitro and in vivo. Mechanistically, XI-001 inhibited the interaction between MDMX and p53, reduced MDMX levels, and stabilized p53 in a dose-dependent manner. Moreover, MDMX colocalizes with E6AP and appears to be a novel binding partner of E6AP that promotes p53 ubiquitination [102]. TBP-like protein (TLP) is a member of the TBP family and can prevent p53 degradation by disrupting the p53-MDM2 interaction. TLP inhibits MDM2-driven nuclear export of p53 to retain it in the nucleus [80]. In terms of physiological significance, TLP can significantly inhibit the growth of solid tumors of the CC in mice. Thus, TLP establishes a novel mechanism for the activation of long-acting p53 to determine cell fate.

#### 6.2.2. Preclinical

Mouse models can be constructed to confirm the tumor suppressive effect of E3-targeting inhibitors, which can be further applied in the clinical stage. However, more studies have focused on fundamental studies of putative targets. In addition, owing to the continuous discovery of the oncogenic role of E3 ligases in EC and CC, the exploration of corresponding specific inhibitors has gradually become a research interest, and some progress has been made, such as targeting E6AP, MDM2, and HUWE1 in CC and targeting SCF^Skp2/Cks1^ and MDM2 in EC. This section focuses on E3 ligase inhibitors of these potential therapeutic targets and their roles in the treatment of EC and CC (Table 4).

As previously described, the E6 oncoprotein expressed in high-risk HPV can regulate p53 levels and transcriptional activity through the ubiquitination pathway in the presence of E6AP [59,207]. Therefore, the design and development of novel anticancer drugs targeting E6AP-p53 have gradually become a research interest. E6AP inhibitors associated with CC include RITA and quercetin [204,208] (Table 4). A study by Zhao et al. confirmed that certain substances that reactivate p53 and induce apoptosis in tumor cells can promote the formation of a ternary complex between p53 and E6/E6AP to protect p53 from ubiquitination and degradation and induce its accumulation [204]. Flavonoids are widely found in most plants. It is commonly associated with various biological activities, including promoting the production of reactive oxygen species (ROS), repairing DNA damage, and restoring the function of p53 [208,209]. Several studies have shown that quercetin, a polyphenolic flavonoid, has anticancer activity against multiple cancers. For example, quercetin induces apoptosis in melanoma cells via a p53/Bax-dependent mechanism while increasing ROS production [200]. Quercetin arrests the cell cycle in the G2 phase and triggers apoptosis in CC cells. Furthermore, quercetin binding to E6 disturbed the interaction between E6 and E6AP [208]. Ultimately, this process suppresses the degradation of p53.

MDM2 can tightly bind to the p53 trans-activation domain and promote ubiquitin-dependent proteasomal degradation [210]. Thus, inhibitors targeting MDM2 may restore the function of p53 and tumor sensitivity to radiotherapy and chemotherapy by disrupting the p53-MDM2 interaction [211]. It is encouraging that some inhibitors targeting MDM2, including idasanutlin, Nutlin-3, and MI-21, have entered the clinical stage for the treatment of PCa [31]. However, the efficacy of these inhibitors in EC and CC remains unclear. Natural products are widely used in clinical practice, and their use has exceeded 50% at present. Tulbaghia violacea (TV) and Agave palmeri (AG) are two plant extracts that act as potential apoptotic inducers in CC. Treatment of CC cells with these two plant extracts has been shown to significantly reduce MDM2 expression; however, the exact biological mechanisms are still unclear [202]. In addition, another MDM2-targeting small-molecule compound, CP-31398, a synthetic styrene quinazoline, was able to down-regulate MDM2 expression and restore p53 mutant function in EC [205].

Several other drugs have been found to regulate substrate degradation by E3 ligases. For example, Androg, a common traditional medicinal plant in Asia, disrupts HERC4 and SMURF2 activities to restore p53 function in HPV16-positive CC cells [201]. Tobramycin can be used as a targeted therapy to prevent or treat HUWE1 overexpressing CC [67]. It specifically binds to the HECT domain and inhibits the degradation of MCL1. In addition, tobramycin regulates the G2/M transition and inhibits the proliferation of CC cells in vitro [67]. Cellular inhibitor of apoptosis protein 1 (CIAP1) is highly expressed in CC and up-regulates resistance to radiotherapy and chemotherapy. Bestatin-methyl ester (ME-BS) directly interacts with CIAP1, promotes its RING domain-dependent autoubiquitination, and promotes proteasomal degradation of CIAP1 [212]. The inhibitory effect of ME-BS on CC provides a novel strategy for manipulating the intrinsic ubiquitin ligase activity. Pavlides et al. developed a novel drug-binding approach to identify the activity of multiple SMIs of SCF^Skp2/Cks1^ E3 ligase (Skp2E3LIs) [206]. Skp2E3LIs stabilize p27 in the nucleus and mediate cell cycle arrest in the G1 phase without cytotoxicity.

### 6.3. Targeting the Substrates of E3 Ligase and Neddylation for EC and CC Therapy

Cullins are the best-characterized neddylation substrates [213]. Currently, advances have been made in exploiting inhibitors for neddylation modifications. An increasing number of studies have confirmed that the small molecule MLN4924 can inactivate CRL function and induce autophagy and apoptosis by blocking the neddylation of Cullin in EC and HCC [214,215]. Some E3 ligases are considered tumor suppressors of EC, and the accumulation of carcinogenic substrates caused by frequent mutations is the cause of cancer. Therefore, the development of corresponding E3 analogs or inhibitors targeting oncogenic substrates may be a neoadjuvant therapy for CC and EC. *FBXW7* mutations increase the phosphorylation of proteins, including SRC-3, cyclin E1, c-Myc, rictor, GSK3, AKT, and P70S6, which are essential causes of EC [153]. Urick et al. provided evidence that two drugs can significantly reduce the survivability of EC cells with *FBXW7* mutant in vitro: SI-2, an SMI of SRC, selectively targets SRC-3; dinaciclib inhibits the activity of Cdk2 as well as Cdk5, 1, and 9 [153]. In addition, the use of adenoviruses as live vectors encoding p53 has shown progressive results in patients with CC. As mentioned above, CUL3^SPOP^ inhibits the progression of EC and PCa by degrading oncoproteins [216]. Interestingly, endometrial CUL3^SPOP^ mutants that sensitize BET inhibitors spontaneously degrade BET proteins; however, PCa-specific CUL3^SPOP^ mutants impair the degradation of BET protein and promote resistance to its pharmacological inhibition [106]. This difference indicates that mutations in the same E3 ligase domain can have different drug sensitivities depending on the tumor.

### 6.4. PROTACs

**Figure 3:**
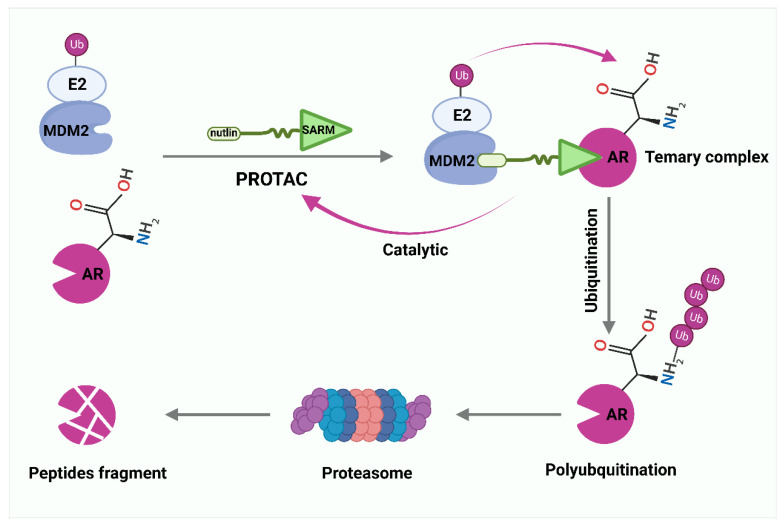
PROTAC-mediated degradation of target proteins through the ubiquitin–proteasome system (UPS). Selective androgen receptor modulator (SARM) and nutlin assemble into a complex that hijacks murine double minute 2 (MDM2). SARM with nutlin binds MDM2 and intracellular androgen receptor (AR), labels AR with ubiquitin, and then induces cells to remove AR by proteasomes.

Inhibitors targeting EC- and CC-related E3 ligases are still at the cellular level and far from the clinical stage. At the same time, the development of these inhibitors has difficulties, such as drug resistance, side effects, and reduced activity against many proteins. Thus, the emergence of new models of proteolysis-targeting chimeras (PROTACs) that control substrate levels provides extraordinary strategies for enhancing drug development [217]. Instead of inhibiting the function of the protein of interest (POI), PROTACs bind an E3 ligase and intracellular POI and label it with ubiquitin, and then induce cells to remove POI by UPS [218]. Importantly, PROTACs can be recycled to exert stronger biological effects [219]. Crews et al. reported the first PROTAC small molecule with high cellular permeability and biostability that can successfully mediate ubiquitination degradation of androgen receptors (ARs) in CC cells at 10Mm [220]. This PROTAC associates a non-steroidal derivative of the selective androgen receptor modulators (SARM) with nutlin to recruit MDM2 (Figure 3). Recently, a new generation of PROTACs targeting AR has been developed, including ARV-110, ARD-266, ARD-61, and ARD-69 [65]. Currently, ARV-110 is entering phase II clinical trials for patients with PCa. Triazolodiazepine-based JQ1 is an inhibitor of Bromo- and Extra-terminal (BET) family of proteins [221]. MZ1 contains two linker ligands, one is JQ1, responsible for recruiting BET proteins, and the other is a von Hippel–Lindau (VHL) ligand [222]. In CC, MZ1 can specifically bind and rapidly degrade BET proteins, especially BRD4 [222]. Similarly, replacing the VHL ligand of MZ1 with the RNF4 ligand leads to another PROTAC drug, CCW28-3 [223]. In addition to MDM2, VHL, and RNF4, cereblon (CRBN), inhibitors of apoptosis (IAPs), DDB1, cul4-related factors (DCAF15 and DCAF16), and RNF114 ligands with PROTACs are offering prospects for the exploration and development of new therapeutic agents [224,225,226,227,228].

## 7. Conclusions and Perspectives

We summarized the relevant mechanisms and mutation sites of these E3 ligases leading to the development and progression of EC and CC. Intervention with these E3 ligases or specific substrates will facilitate subsequent treatment.

Several SMIs and activators, such as bortezomib, Nutlin, and RITA, have been developed to treat various diseases caused by UPS dysfunction. These SMIs have the advantages of low molecular weight, high cell/tissue permeability, high oral bioavailability, and little off-target. In addition, SMIs are more tolerant to target proteins that maintain normal cell function. However, these SMIs are prone to drug tolerance and cytotoxicity due to the need for frequent dosing. An example is bortezomib-induced peripheral neuropathy. The efficacy of these SMIs requires a high affinity for the target protein, so they can hardly completely inhibit the function of their target. Currently, most of the efficacy testing of SMIs is stalled at the cellular level. This is unfavorable for subsequent clinical applications. Therefore, animal experiments, such as the construction of the mouse Pdx model, can be further verified its efficacy. XI-001 and TLP successfully inhibited the growth of CC tissue in xenograft mice, but their toxicities are currently unknown. For SMIs used in the clinic, toxic side effects, specific affinity to target proteins, and drug absorption need to be further detected.

The development of SMIs is the primary strategy for targeted therapy of E3 ligase dysfunction. However, most SMIs developed have poor potency and selectivity. Therefore, understanding the action mechanism of E3 ligases and the toxicity mechanism of SMIs is critical for drug screening and discovery. The innovation of PROTAC technology solves various difficulties in developing targeted E3 ligase inhibitors and opens a new stage in research and new drug development. However, identifying the best ligand capable of designing PROTAC remains a challenge. Although numerous E3 ligases have been identified, only some can be recruited into chimeric small molecules to degrade target proteins, such as FBXW7, MDM2, VHL, and KEAP1. In addition, several novel PROTACs drugs have been successfully designed and applied to the degradation of oncogenic proteins in CC cells, such as MZI and CCW28-3. Whether they can be applied to the degradation of the same oncogenic proteins in EC cells remains unknown. It may be related to the tissue specificity of oncogenic proteins. Whether the E3 ligases associated with EC and CC summarized in this review can act as ligands for PROTAC also remains unknown. This review also facilitates the search for PROTAC ligands for further explorers. Therefore, we have reason to believe that these PROTACs have promise for the treatment of EC and CC in the future.

E3 ligase dysfunction is essential in the development and progression of EC and CC. By focusing on the development of more targeted SMIs or activators of E3 ligases, the therapeutic effect will be significantly improved, which will also meet the diagnosis and treatment requirements of cancer precision medicine.

## Figures and Tables

**Table 1:** Functional roles of E3 ligases in EC and CC.

E3 Ligase	Targets for Ubiquitination	Degraded or Not	Effect	Reference
p53 Pathway				
MDM2	p53	Yes	Promote the occurrence and development of EC and CC	[57,58]
E6AP	p53	Yes	Promote the occurrence and development of CC	[59]
TRIM65	p53	Yes	Suppress apoptosis of CC cells	[13]
RBBP6	p53	Yes	Promote the migration and proliferation of CC cells	[60]
NF-κB pathway				
CUL3^SPOP^	DRAK1	Yes	Promoting the progression of CC	[61]
TRIM22	NOD2	NO	Suppress progress of EC patients	[62]
TRIM25	14-3-3σ	Yes	Promote the progression of EC	[63]
EGFR pathway				
MKRN1	PTEN	Yes	Promote the progression of CC	[64]
Wnt/β-catenin pathway				
RNF43	Wnt	Yes	Suppress progress of EC patients	[65]
TGF-β/Smad pathway				
SCF^Skp2/Cks1^	p27	Yes	Promote the growth of EC cell	[66]
Other E3 ligases				
SCF^FBXO2^	Fibrillin	Yes	Promote cell cycle and autophagy of EC	[67]
SCF^FBXW7^	CCNE1	Yes	Promote the proliferation, growth, and apoptosis of EC	[68]
APC^Cdc20^	p21	Yes	Suppress the occurrence and development of EC	[69]
HUWE1	c-Myc	Yes	Promote the proliferation of CC	[70]
MDM2	IER3	Yes	Suppress the apoptosis of CC cells	[71]
RNF114	PARP10	NO	Suppress the EMT and metastasis of tumor cells	[72]
Smurf1	FOXA2	Yes	Promote metastasis of CC cells	[73]
NEDD4L	Unknown	Unknown	Promote the occurrence and development of EC	[74]

**Table 2:** Type and site of mutations in EC.

E3	Mutation Frequency	Missense			Truncating	Others
		Activated	Inactivated	Undetected		
MDM2	2.4%	-	-	E151D, R189C, S259Y, R71Q, S413N, D385G, F297S, R169I, W335C, S268G, A440V, T16A, S226L	L321Ffs*52	X280_splice
TRIM65	0.8%	-	-	L409P, T196M	-	-
RBBP6	9.1%	-	-	K1735N, P1067L, T326A, R755Q, T1452M, K180N, N214S, E1330K, A95S, R1433C, P338H, R1290Q, S1662Y, R826H, A457T, D817A, E1518D, L369P, S1262I, A496V	R1290*, E1654*, R755*, E650*, E1230*, R892*, E1284*, R1497*, Q483*, K1138Hfs*18, P1501Ffs*4	X318_splice, X225_splice, X146_splice, L332del, K1112del
CUL3^SPOP^	8.7%	M117V, R121Q, E50K	D140G	D140N, W131C, E47A, E78K, S14L, S14L, R354H, R45W, L282R	R240*, R70*, R370Tfs*12, S59*	-
TRIM22	3.7%	-	-	S312L, R132H, F469L, A341T, S383R, K332N, F457V, K138N, G186E	W357*, R279*, C116*, E24*	-
TRIM25	2.1%	-	-	K388N, L281I, S286N, A219S	C13*	-
RNF43	5.0%	-	-	R531H, S607L, P154L, R286W, R219H, P154S, S85F, P587S	R337*, R132*, R519*, Q22*, W302Cfs*117, G659Vfs*41	-
SCF^Skp2^	2.5%	-	-	R154Q, R182C, R217Q, T225I, V183I, H273R		-
SCF^FBXO2^	0.4%	-	-	N229T		
SCF^FBXW7^	15.7%	-	R465H, R479Q, R465C, R505C	R658Q, Y545C, R505G, R689W, G423V, R224Q, R689Q, R441Q, E248D, S476I, H382N, V418M, P247T, L320I, D135Y, H52Y, D129A	R224*, R393*, R658*, R13*, R14*, M268Dfs*18, E369*, W237*	X329_splice
APC^Cdc20^	1.2%	-	-	R262Q, V426F	W276*	-
HUWE1	11.2%	-	-	A1545V, D1327Y, P2947L, R2214W, R3178W, R3071H, P4076H, D2706N, L3923S, M1001V, R3365W, R3957C, L2658I, F1537C, A1499D, E85D, N1890S, S3513F, L3212I, S2842Y, K2759N, E2295K, D787Y, K2Q, T4303I, P3981H, A2174T, A2025T, D1773Y, P695H, L556I, L93F, A4363V, L601I, K3742N, N783S, S327Y, T785I, S3461L, S4148I, L466S, S4084P, K3837N, G1904E, D1753Y, R3111H, V2308A, V933I, D593Y, R3998H, P3699S, L2658F, G2208D, R1780H, T3737M, K3621E, S4287Y, L3733I, K2043N, V131M, S3470L, R3297S, R2838K, M2465T, L1823F, E4006D, G3177E, G1749V, D3813E, T1214M, A4058V, I2212V	E1709*, E4040*, E117*, E1435*, S2327*, G1904*	S3794=, S990=
RNF114	1.2%	-	-	A70T, R68H, K61N		-
Smurf1	4.5%	-	-	R218Q, A561T, E375K, A665V, V659M, D377Y, A396V, D110Y, R494Q, E284K, T245A	R218*	-
NEDD4L	3.7%	-	-	E893K, R400C, R895H, R633Q, P949H, E674D, T375M, R124Q, R468W, S256R, A33T	-	-

**Table 3:** Type and site of mutations in CC.

E3	Mutation Frequency	Missense
MDM2	0.5%	L91V
TRIM65	1.9%	R331C
RBBP6	3.7%	A846V, N931S, N1649D
RNF43	0.9%	M522L
Skp2	0.9%	H148N
HUWE1	0.9%	G2872V
NEDD4L	0.9%	R248C

**Table 4:** Summary of inhibitors targeting the E3s in EC and CC.

Targets	Compound	Mechanism of Action	Clinical Stage in HCC	Reference
Plant Extract				
E6AP	Quercetin	Non-competitive Inhibit the formation of E6/E6AP/p53 ternary complex	Preclinical	[200]
HERC4	Androg	Disrupt the activity of HERC4	Preclinical	[201]
SMURF2	Androg	Disrupt the activity of SMURF2	Preclinical	[201]
MDM2	TV/AG	Downregulate the expression of MDM2	Preclinical	[202]
Synthesis				
MDM2	Nutlin	Promote the degradation of AR	Preclinical	[203]
E6AP	RITA	Non-competitive inhibit the formation of E6/E6AP/p53 ternary complex	Preclinical	[204]
MDM2	CP-31398	Block MDM2 and p53 interaction	Preclinical	[205]
SCF^Skp2/Cks^	Skp2E3LIs	Competitively inhibit the binding of SCF^Skp2/Cks^ to p27 and mediate p27 accumulation	Preclinical	[206]

## References

[1] Azadehrah M., Vosoogh S., Azadehrah M. (2022). The roles and therapeutic applications of cytokines in endometrial cancer. J. Reprod. Immunol..

[2] Ravegnini G., Gorini F., De Crescenzo E., De Leo A., De Biase D., Di Stanislao M., Hrelia P., Angelini S., De Iaco P., Perrone A.M. (2022). Can miRNAs be useful biomarkers in improving prognostic stratification in endometrial cancer patients? An update review. Int. J. Cancer.

[3] Siegel R.L., Miller K.D., Fuchs H.E., Jemal A. (2022). Cancer statistics, 2022. CA Cancer J. Clin..

[4] Matsuura M., Suzuki T., Morishita M., Tanaka R., Ito E., Saito T. (2009). Chemotherapy (CT) with radiotherapy versus CT alone for FIGO Stage IIIc endometrial cancer. Eur. J. Gynaecol. Oncol..

[5] Talhouk A., McConechy M.K., Leung S., Yang W., Lum A., Senz J., Boyd N., Pike J., Anglesio M., Kwon J.S. (2017). Confirmation of ProMisE: A simple, genomics-based clinical classifier for endometrial cancer. Cancer.

[6] Mandato V.D., Mastrofilippo V., Palicelli A., Silvotti M., Serra S., Giaccherini L., Aguzzoli L. (2021). Solitary vulvar metastasis from early-stage endometrial cancer: Case report and literature review. Medicine.

[7] Bokhman J.V. (1983). Two pathogenetic types of endometrial carcinoma. Gynecol. Oncol..

[8] Mandato V.D., Palicelli A., Torricelli F., Mastrofilippo V., Leone C., Dicarlo V., Tafuni A., Santandrea G., Annunziata G., Generali M. (2022). Should Endometrial Cancer Treatment Be Centralized?. Biology.

[9] Kandoth C., Schultz N., Cherniack A.D., Akbani R., Liu Y., Shen H., Robertson A.G., Pashtan I., Shen R., Benz C.C. (2013). Integrated genomic characterization of endometrial carcinoma. Nature.

[10] Jemal A., Bray F., Center M.M., Ferlay J., Ward E., Forman D. (2011). Global cancer statistics. CA Cancer J. Clin..

[11] Ferlay J., Shin H.R., Bray F., Forman D., Mathers C., Parkin D.M. (2010). Estimates of worldwide burden of cancer in 2008: GLOBOCAN 2008. Int. J. Cancer.

[12] Rossman A.H., Reid H.W., Pieters M.M., Mizelle C., von Isenburg M., Ramanujam N., Huchko M.J., Vasudevan L. (2021). Digital Health Strategies for Cervical Cancer Control in Low- and Middle-Income Countries: Systematic Review of Current Implementations and Gaps in Research. J. Med. Internet Res..

[13] Wang X.Y., Mao H.W., Guan X.H., Huang Q.M., Yu Z.P., Wu J., Tan H.L., Zhang F., Huang X., Deng K.Y. (2022). TRIM65 Promotes Cervical Cancer Through Selectively Degrading p53-Mediated Inhibition of Autophagy and Apoptosis. Front. Oncol..

[14] Lou Z., Wang S. (2014). E3 ubiquitin ligases and human papillomavirus-induced carcinogenesis. J. Int. Med. Res..

[15] Mammas I.N., Sourvinos G., Giannoudis A., Spandidos D.A. (2008). Human papilloma virus (HPV) and host cellular interactions. Pathol. Oncol. Res..

[16] Subramanian P.D., An Z., Yu J.R., Park W.Y. (2016). Silencing of Fused Toes Homolog Enhances Cisplatin Sensitivity in Cervical Cancer Cells by Inhibiting Epidermal Growth Factor Receptor-Mediated Repair of DNA Damage. Cancer Chemother. Pharmacol..

[17] Prabakaran D.S., Chaturvedi P.K., Krishnamoorthy D., Seo Y.S., Thippana M., Park W.Y. (2022). Fused Toes Homolog, a Potential Molecular Regulator of Human Papillomavirus Type 16 E6 and E7 Oncoproteins in Cervical Cancer. PLoS ONE.

[18] Prabakaran D.S., Chaturvedi P.K., Shimokawa T., Kim K.H., Park W.Y. (2021). Silencing of Fused Toes Homolog (Fts) Increases Radiosensitivity to Carbon-Ion through Downregulation of Notch Signaling in Cervical Cancer Cells. Front. Oncol..

[19] Muthusami S., Prabakaran D.S., Yu J.R., Park W.Y. (2015). Fts Is Responsible for Radiation-Induced Nuclear Phosphorylation of Egfr and Repair of DNA Damage in Cervical Cancer Cells. J. Cancer Res. Clin. Oncol..

[20] Prabakaran D.S., Muthusami S., Sivaraman T., Yu J.R., Park W.Y. (2019). Silencing of Fts Increases Radiosensitivity by Blocking Radiation-Induced Notch1 Activation and Spheroid Formation in Cervical Cancer Cells. Int. J. Biol. Macromol..

[21] Muthusami S., Prabakaran D.S., Yu J.R., Park W.Y. (2014). Egf-Induced Expression of Fused Toes Homolog (Fts) Facilitates Epithelial-Mesenchymal Transition and Promotes Cell Migration in Me180 Cervical Cancer Cells. Cancer Lett..

[22] Ikeda F., Dikic I. (2008). Atypical ubiquitin chains: New molecular signals. ‘Protein Modifications: Beyond the Usual Suspects’ review series. EMBO Rep..

[23] Mukhopadhyay D., Riezman H. (2007). Proteasome-independent functions of ubiquitin in endocytosis and signaling. Science.

[24] Senft D., Qi J., Ronai Z.A. (2018). Ubiquitin ligases in oncogenic transformation and cancer therapy. Nat. Rev. Cancer.

[25] Morrow J.K., Lin H.K., Sun S.C., Zhang S. (2015). Targeting ubiquitination for cancer therapies. Future Med. Chem..

[26] Zheng N., Shabek N. (2017). Ubiquitin Ligases: Structure, Function, and Regulation. Annu. Rev. Biochem..

[27] Fajner V., Maspero E., Polo S. (2017). Targeting HECT-type E3 ligases—Insights from catalysis, regulation and inhibitors. FEBS Lett..

[28] Berndsen C.E., Wolberger C. (2014). New insights into ubiquitin E3 ligase mechanism. Nat. Struct. Mol. Biol..

[29] Dove K.K., Klevit R.E. (2017). RING-Between-RING E3 Ligases: Emerging Themes amid the Variations. J. Mol. Biol..

[30] Guo J., Wu Y., Du J., Yang L., Chen W., Gong K., Dai J., Miao S., Jin D., Xi S. (2018). Deregulation of UBE2C-mediated autophagy repression aggravates NSCLC progression. Oncogenesis.

[31] Zhao Y., Li J., Chen J., Ye M., Jin X. (2022). Functional roles of E3 ubiquitin ligases in prostate cancer. J. Mol. Med..

[32] Li K., Li J., Ye M., Jin X. (2022). The role of Siah2 in tumorigenesis and cancer therapy. Gene.

[33] Huibregtse J.M., Scheffner M., Beaudenon S., Howley P.M. (1995). A family of proteins structurally and functionally related to the E6-AP ubiquitin-protein ligase. Proc. Natl. Acad. Sci. USA.

[34] Scheffner M., Nuber U., Huibregtse J.M. (1995). Protein ubiquitination involving an E1-E2-E3 enzyme ubiquitin thioester cascade. Nature.

[35] Sluimer J., Distel B. (2018). Regulating the human HECT E3 ligases. Cell. Mol. Life Sci..

[36] Rotin D., Kumar S. (2009). Physiological functions of the HECT family of ubiquitin ligases. Nat. Rev. Mol. Cell Biol..

[37] Dunn R., Klos D.A., Adler A.S., Hicke L. (2004). The C2 domain of the Rsp5 ubiquitin ligase binds membrane phosphoinositides and directs ubiquitination of endosomal cargo. J. Cell Biol..

[38] Tian M., Bai C., Lin Q., Lin H., Liu M., Ding F., Wang H.R. (2011). Binding of RhoA by the C2 domain of E3 ligase Smurf1 is essential for Smurf1-regulated RhoA ubiquitination and cell protrusive activity. FEBS Lett..

[39] Rizo J., Südhof T.C. (1998). C2-domains, structure and function of a universal Ca^2+^-binding domain. J. Biol. Chem..

[40] Bischoff F.R., Ponstingl H. (1991). Catalysis of guanine nucleotide exchange on Ran by the mitotic regulator RCC1. Nature.

[41] Zhang C., Clarke P.R. (2000). Chromatin-independent nuclear envelope assembly induced by Ran GTPase in Xenopus egg extracts. Science.

[42] Nemergut M.E., Mizzen C.A., Stukenberg T., Allis C.D., Macara I.G. (2001). Chromatin docking and exchange activity enhancement of RCC1 by histones H2A and H2B. Science.

[43] Lemak A., Yee A., Bezsonova I., Dhe-Paganon S., Arrowsmith C.H. (2011). Zn-binding AZUL domain of human ubiquitin protein ligase Ube3A. J. Biomol. NMR.

[44] Scheffner M., Kumar S. (2014). Mammalian HECT ubiquitin-protein ligases: Biological and pathophysiological aspects. Biochim. Biophys. Acta.

[45] Freemont P.S., Hanson I.M., Trowsdale J. (1991). A novel cysteine-rich sequence motif. Cell.

[46] Deshaies R.J., Joazeiro C.A. (2009). RING domain E3 ubiquitin ligases. Annu. Rev. Biochem..

[47] Bulatov E., Ciulli A. (2015). Targeting Cullin-RING E3 ubiquitin ligases for drug discovery: Structure, assembly and small-molecule modulation. Biochem. J..

[48] Ozkan E., Yu H., Deisenhofer J. (2005). Mechanistic insight into the allosteric activation of a ubiquitin-conjugating enzyme by RING-type ubiquitin ligases. Proc. Natl. Acad. Sci. USA.

[49] Liu B., Tang Y., Yang P., Wu C., Huang Y. (2021). TRIM65 in White Matter Lesions, Innate Immunity, and Tumor. Curr. Mol. Pharmacol..

[50] Aguilera M., Oliveros M., Martínez-Padrón M., Barbas J.A., Ferrús A. (2000). Ariadne-1: A vital Drosophila gene is required in development and defines a new conserved family of ring-finger proteins. Genetics.

[51] Ryu M.Y., Cho S.K., Hong Y., Kim J., Kim J.H., Kim G.M., Chen Y.J., Knoch E., Møller B.L., Kim W.T. (2019). Classification of barley U-box E3 ligases and their expression patterns in response to drought and pathogen stresses. BMC Genom..

[52] Jin X., Qing S., Li Q., Zhuang H., Shen L., Li J., Qi H., Lin T., Lin Z., Wang J. (2021). Prostate cancer-associated SPOP mutations lead to genomic instability through disruption of the SPOP-HIPK2 axis. Nucleic Acids Res..

[53] Hatakeyama S., Yada M., Matsumoto M., Ishida N., Nakayama K.I. (2001). U box proteins as a new family of ubiquitin-protein ligases. J. Biol. Chem..

[54] Murata S., Chiba T., Tanaka K. (2003). CHIP: A quality-control E3 ligase collaborating with molecular chaperones. Int. J. Biochem. Cell Biol..

[55] Sarkar S., Brautigan D.L., Larner J.M. (2017). Aurora Kinase A Promotes AR Degradation via the E3 Ligase CHIP. Mol. Cancer Res..

[56] Sarkar S., Brautigan D.L., Parsons S.J., Larner J.M. (2014). Androgen receptor degradation by the E3 ligase CHIP modulates mitotic arrest in prostate cancer cells. Oncogene.

[57] Bose I., Ghosh B. (2007). The p53-MDM2 network: From oscillations to apoptosis. J. Biosci..

[58] Mendoza M., Mandani G., Momand J. (2014). The MDM2 gene family. Biomol. Concepts.

[59] Li S., Hong X., Wei Z., Xie M., Li W., Liu G., Guo H., Yang J., Wei W., Zhang S. (2019). Ubiquitination of the HPV Oncoprotein E6 Is Critical for E6/E6AP-Mediated p53 Degradation. Front. Microbiol..

[60] Teng F., Ruan H.J., Xu J., Ni J., Qian B., Shen R., Gao L.J. (2018). RBBP6 promotes human cervical carcinoma malignancy via JNK signaling pathway. Biomed. Pharmacother..

[61] Park Y., Pang K., Park J., Hong E., Lee J., Ooshima A., Kim H.S., Cho J.H., Han Y., Lee C. (2020). Destablilization of TRAF6 by DRAK1 Suppresses Tumor Growth and Metastasis in Cervical Cancer Cells. Cancer Res..

[62] Zhang L., Zhang B., Wei M., Xu Z., Kong W., Deng K., Xu X., Zhang L., Ζhao X., Yan L. (2020). TRIM22 inhibits endometrial cancer progression through the NOD2/NF-κB signaling pathway and confers a favorable prognosis. Int. J. Oncol..

[63] Sato W., Ikeda K., Urano T., Abe Y., Nakasato N., Horie-Inoue K., Takeda S., Inoue S. (2018). Efp promotes in vitro and in vivo growth of endometrial cancer cells along with the activation of nuclear factor-κB signaling. PLoS ONE.

[64] Lee M.S., Jeong M.H., Lee H.W., Han H.J., Ko A., Hewitt S.M., Kim J.H., Chun K.H., Chung J.Y., Lee C. (2015). Pi3k/Akt Activation Induces Pten Ubiquitination and Destabilization Accelerating Tumourigenesis. Nat. Commun..

[65] Han X., Zhao L., Xiang W., Qin C., Miao B., Xu T., Wang M., Yang C.Y., Chinnaswamy K., Stuckey J. (2019). Discovery of Highly Potent and Efficient PROTAC Degraders of Androgen Receptor (AR) by Employing Weak Binding Affinity VHL E3 Ligase Ligands. J. Med. Chem..

[66] Di Cristofano A., Ellenson L.H. (2007). Endometrial carcinoma. Annu. Rev. Pathol..

[67] He J., Wu S., Li X., Tang L., Chen H., Qin L., Xie J., Lu T., Xu W. (2020). Tobramycin suppresses HUWE1 degradation to control MCL-1 stability during tumour development. Clin. Exp. Pharmacol. Physiol..

[68] Kuhn E., Wu R.C., Guan B., Wu G., Zhang J., Wang Y., Song L., Yuan X., Wei L., Roden R.B. (2012). Identification of molecular pathway aberrations in uterine serous carcinoma by genome-wide analyses. J. Natl. Cancer Inst..

[69] Kong D., Hou Y., Li W., Ma X., Jiang J. (2022). LncRNA-ZXF1 stabilizes P21 expression in endometrioid endometrial carcinoma by inhibiting ubiquitination-mediated degradation and regulating the miR-378a-3p/PCDHA3 axis. Mol. Oncol..

[70] Bonazzoli E., Bellone S., Zammataro L., Gnutti B., Guglielmi A., Pelligra S., Nagarkatti N., Manara P., Tymon-Rosario J., Zeybek B. (2020). Derangements in HUWE1/c-MYC pathway confer sensitivity to the BET bromodomain inhibitor GS-626510 in uterine cervical carcinoma. Gynecol. Oncol..

[71] Jin H., Lee K., Kim Y.H., Oh H.K., Maeng Y.I., Kim T.H., Suh D.S., Bae J. (2016). Scaffold protein FHL2 facilitates MDM2-mediated degradation of IER3 to regulate proliferation of cervical cancer cells. Oncogene.

[72] Zhao Y., Liang X., Wei L., Liu Y., Liu J., Feng H., Zheng F., Wang Y., Ma H., Wu J. (2021). RNF114 suppresses metastasis through regulation of PARP10 in cervical cancer cells. Cancer Commun..

[73] Zhan F., Zhong Y., Qin Y., Li L., Wu W., Yao M. (2020). SND1 facilitates the invasion and migration of cervical cancer cells by Smurf1-mediated degradation of FOXA2. Exp. Cell Res..

[74] Yilmaz E., Gul M., Melekoglu R., Inci Coskun E., Sahin N., Gul S., Bastemur A.G., Ciplak B. (2018). Neural precursor cell-expressed developmentally down-regulated 4-like: A new biomarker in the pathophysiology of endometrial cancer. J. Int. Med. Res..

[75] Vogelstein B., Lane D., Levine A.J. (2000). Surfing the p53 network. Nature.

[76] Wei C.L., Wu Q., Vega V.B., Chiu K.P., Ng P., Zhang T., Shahab A., Yong H.C., Fu Y., Weng Z. (2006). A global map of p53transcription-factor binding sites in the human genome. Cell.

[77] Geyer R.K., Yu Z.K., Maki C.G. (2000). The MDM2 RING-finger domain is required to promote p53 nuclear export. Nat. Cell Biol..

[78] Nie L., Sasaki M., Maki C.G. (2007). Regulation of p53 nuclear export through sequential changes in conformation and ubiquitination. J. Biol. Chem..

[79] Carter S., Bischof O., Dejean A., Vousden K.H. (2007). C-terminal modifications regulate MDM2 dissociation and nuclear export of p53. Nat. Cell Biol..

[80] Maeda R., Tamashiro H., Takano K., Takahashi H., Suzuki H., Saito S., Kojima W., Adachi N., Ura K., Endo T. (2017). TBP-like Protein (TLP) Disrupts the p53-MDM2 Interaction and Induces Long-lasting p53 Activation. J. Biol. Chem..

[81] Koo N., Sharma A.K., Narayan S. (2022). Therapeutics Targeting p53-MDM2 Interaction to Induce Cancer Cell Death. Int. J. Mol. Sci..

[82] Inoue K., Fry E.A., Frazier D.P. (2016). Transcription factors that interact with p53 and Mdm2. Int. J. Cancer.

[83] Shaikh M.F., Morano W.F., Lee J., Gleeson E., Babcock B.D., Michl J., Sarafraz-Yazdi E., Pincus M.R., Bowne W.B. (2016). Emerging Role of MDM2 as Target for Anti-Cancer Therapy: A Review. Ann. Clin. Lab. Sci..

[84] Feng J., Tamaskovic R., Yang Z., Brazil D.P., Merlo A., Hess D., Hemmings B.A. (2004). Stabilization of Mdm2 via decreased ubiquitination is mediated by protein kinase B/Akt-dependent phosphorylation. J. Biol. Chem..

[85] Mayo L.D., Donner D.B. (2001). A phosphatidylinositol 3-kinase/Akt pathway promotes translocation of Mdm2 from the cytoplasm to the nucleus. Proc. Natl. Acad. Sci. USA.

[86] Wu X., Bayle J.H., Olson D., Levine A.J. (1993). The p53-mdm-2 autoregulatory feedback loop. Genes Dev..

[87] Liu L., Yang L., Chang H., Chen Y.N., Zhang F., Feng S., Peng J., Ren C.C., Zhang X.A. (2019). CP-31398 attenuates endometrial cancer cell invasion, metastasis and resistance to apoptosis by downregulating MDM2 expression. Int. J. Oncol..

[88] Vogel R.I., Pulver T., Heilmann W., Mooneyham A., Mullany S., Zhao X., Shahi M., Richter J., Klein M., Chen L. (2016). USP14 is a predictor of recurrence in endometrial cancer and a molecular target for endometrial cancer treatment. Oncotarget.

[89] He X., Kong X., Yan J., Yan J., Zhang Y., Wu Q., Chang Y., Shang H., Dou Q., Song Y. (2015). CP-31398 prevents the growth of p53-mutated colorectal cancer cells in vitro and in vivo. Tumor Biol..

[90] Fiorini C., Menegazzi M., Padroni C., Dando I., Dalla Pozza E., Gregorelli A., Costanzo C., Palmieri M., Donadelli M. (2013). Autophagy induced by p53-reactivating molecules protects pancreatic cancer cells from apoptosis. Apoptosis.

[91] Schwarz S.E., Rosa J.L., Scheffner M. (1998). Characterization of human hect domain family members and their interaction with UbcH5 and UbcH7. J. Biol. Chem..

[92] Huibregtse J.M., Scheffner M., Howley P.M. (1993). Cloning and expression of the cDNA for E6-AP, a protein that mediates the interaction of the human papillomavirus E6 oncoprotein with p53. Mol. Cell. Biol..

[93] Bernard X., Robinson P., Nominé Y., Masson M., Charbonnier S., Ramirez-Ramos J.R., Deryckere F., Travé G., Orfanoudakis G. (2011). Proteasomal degradation of p53 by human papillomavirus E6 oncoprotein relies on the structural integrity of p53 core domain. PLoS ONE.

[94] Vats A., Skrabar N., Del Sal G., Banks L. (2022). Loss of the E6AP Ubiquitin Ligase Induces p53-Dependent Phosphorylation of Human Papillomavirus 18 E6 in Cells Derived from Cervical Cancer. J. Virol..

[95] Simons A., Melamed-Bessudo C., Wolkowicz R., Sperling J., Sperling R., Eisenbach L., Rotter V. (1997). PACT: Cloning and characterization of a cellular p53 binding protein that interacts with Rb. Oncogene.

[96] Gao S., Scott R.E. (2002). P2P-R protein overexpression restricts mitotic progression at prometaphase and promotes mitotic apoptosis. J. Cell. Physiol..

[97] Mbita Z., Meyer M., Skepu A., Hosie M., Rees J., Dlamini Z. (2012). De-regulation of the RBBP6 isoform 3/DWNN in human cancers. Mol. Cell. Biochem..

[98] Pugh D.J., Ab E., Faro A., Lutya P.T., Hoffmann E., Rees D.J. (2006). DWNN, a novel ubiquitin-like domain, implicates RBBP6 in mRNA processing and ubiquitin-like pathways. BMC Struct. Biol..

[99] Chibi M., Meyer M., Skepu A., DJ G.R., Moolman-Smook J.C., Pugh D.J. (2008). RBBP6 interacts with multifunctional protein YB-1 through its RING finger domain, leading to ubiquitination and proteosomal degradation of YB-1. J. Mol. Biol..

[100] Li L., Deng B., Xing G., Teng Y., Tian C., Cheng X., Yin X., Yang J., Gao X., Zhu Y. (2007). PACT is a negative regulator of p53 and essential for cell growth and embryonic development. Proc. Natl. Acad. Sci. USA.

[101] Hayden M.S., Ghosh S. (2012). NF-κB, the first quarter-century: Remarkable progress and outstanding questions. Genes Dev..

[102] Zhang J., Yu G., Yang Y., Wang Y., Guo M., Yin Q., Yan C., Tian J., Fu F., Wang H. (2022). A small-molecule inhibitor of MDMX suppresses cervical cancer cells via the inhibition of E6-E6AP-p53 axis. Pharmacol. Res..

[103] Pikarsky E., Porat R.M., Stein I., Abramovitch R., Amit S., Kasem S., Gutkovich-Pyest E., Urieli-Shoval S., Galun E., Ben-Neriah Y. (2004). NF-kappaB functions as a tumour promoter in inflammation-associated cancer. Nature.

[104] Hayden M.S., Ghosh S. (2008). Shared principles in NF-kappaB signaling. Cell.

[105] Zhang P., Gao K., Jin X., Ma J., Peng J., Wumaier R., Tang Y., Zhang Y., An J., Yan Q. (2015). Endometrial cancer-associated mutants of SPOP are defective in regulating estrogen receptor-α protein turnover. Cell Death Dis..

[106] Janouskova H., El Tekle G., Bellini E., Udeshi N.D., Rinaldi A., Ulbricht A., Bernasocchi T., Civenni G., Losa M., Svinkina T. (2017). Opposing effects of cancer-type-specific SPOP mutants on BET protein degradation and sensitivity to BET inhibitors. Nat. Med..

[107] Cuneo M.J., Mittag T. (2019). The ubiquitin ligase adaptor SPOP in cancer. FEBS J..

[108] Zhou W., Slingerland J.M. (2014). Links between oestrogen receptor activation and proteolysis: Relevance to hormone-regulated cancer therapy. Nat. Rev. Cancer.

[109] Geng C., He B., Xu L., Barbieri C.E., Eedunuri V.K., Chew S.A., Zimmermann M., Bond R., Shou J., Li C. (2013). Prostate cancer-associated mutations in speckle-type POZ protein (SPOP) regulate steroid receptor coactivator 3 protein turnover. Proc. Natl. Acad. Sci. USA.

[110] Pang K., Lee J., Kim J., Park J., Park Y., Hong E., An H., Ooshima A., Son M., Park K.S. (2022). Degradation of DRAK1 by CUL3/SPOP E3 Ubiquitin ligase promotes tumor growth of paclitaxel-resistant cervical cancer cells. Cell Death Dis..

[111] Duan Z., Gao B., Xu W., Xiong S. (2008). Identification of TRIM22 as a RING finger E3 ubiquitin ligase. Biochem. Biophys. Res. Commun..

[112] Sun Y., Ho G.H., Koong H.N., Sivaramakrishnan G., Ang W.T., Koh Q.M., Lin V.C. (2013). Down-regulation of tripartite-motif containing 22 expression in breast cancer is associated with a lack of p53-mediated induction. Biochem. Biophys. Res. Commun..

[113] Hatakeyama S. (2011). TRIM proteins and cancer. Nat. Rev. Cancer.

[114] Wittmann S., Wunder C., Zirn B., Furtwängler R., Wegert J., Graf N., Gessler M. (2008). New prognostic markers revealed by evaluation of genes correlated with clinical parameters in Wilms tumors. Genes Chromosom. Cancer.

[115] Watanabe T., Asano N., Meng G., Yamashita K., Arai Y., Sakurai T., Kudo M., Fuss I.J., Kitani A., Shimosegawa T. (2014). NOD2 downregulates colonic inflammation by IRF4-mediated inhibition of K63-linked polyubiquitination of RICK and TRAF6. Mucosal Immunol..

[116] Watanabe T., Asano N., Murray P.J., Ozato K., Tailor P., Fuss I.J., Kitani A., Strober W. (2008). Muramyl dipeptide activation of nucleotide-binding oligomerization domain 2 protects mice from experimental colitis. J. Clin. Investig..

[117] Saito-Kanatani M., Urano T., Hiroi H., Momoeda M., Ito M., Fujii T., Inoue S. (2015). Identification of TRIM22 as a progesterone-responsive gene in Ishikawa endometrial cancer cells. J. Steroid Biochem. Mol. Biol..

[118] Inoue S., Orimo A., Hosoi T., Kondo S., Toyoshima H., Kondo T., Ikegami A., Ouchi Y., Orimo H., Muramatsu M. (1993). Genomic binding-site cloning reveals an estrogen-responsive gene that encodes a RING finger protein. Proc. Natl. Acad. Sci. USA.

[119] Urano T., Saito T., Tsukui T., Fujita M., Hosoi T., Muramatsu M., Ouchi Y., Inoue S. (2002). Efp targets 14-3-3 sigma for proteolysis and promotes breast tumour growth. Nature.

[120] Gack M.U., Shin Y.C., Joo C.H., Urano T., Liang C., Sun L., Takeuchi O., Akira S., Chen Z., Inoue S. (2007). TRIM25 RING-finger E3 ubiquitin ligase is essential for RIG-I-mediated antiviral activity. Nature.

[121] Gack M.U., Albrecht R.A., Urano T., Inn K.S., Huang I.C., Carnero E., Farzan M., Inoue S., Jung J.U., García-Sastre A. (2009). Influenza A virus NS1 targets the ubiquitin ligase TRIM25 to evade recognition by the host viral RNA sensor RIG-I. Cell Host Microbe.

[122] Nakayama H., Sano T., Motegi A., Oyama T., Nakajima T. (2005). Increasing 14-3-3 sigma expression with declining estrogen receptor alpha and estrogen-responsive finger protein expression defines malignant progression of endometrial carcinoma. Pathol. Int..

[123] Dai H., Zhao S., Xu L., Chen A., Dai S. (2010). Expression of Efp, VEGF and bFGF in normal, hyperplastic and malignant endometrial tissue. Oncol. Rep..

[124] Cully M., You H., Levine A.J., Mak T.W. (2006). Beyond Pten Mutations: The Pi3k Pathway as an Integrator of Multiple Inputs during Tumorigenesis. Nat. Rev. Cancer.

[125] Chen Y., Shao X., Cao J., Zhu H., Yang B., He Q., Ying M. (2021). Phosphorylation Regulates Cullin-Based Ubiquitination in Tumorigenesis. Acta Pharm. Sin. B.

[126] Behrens J., von Kries J.P., Kühl M., Bruhn L., Wedlich D., Grosschedl R., Birchmeier W. (1996). Functional interaction of beta-catenin with the transcription factor LEF-1. Nature.

[127] Chen L., Xu Z., Li Q., Feng Q., Zheng C., Du Y., Yuan R., Peng X. (2021). USP28 facilitates pancreatic cancer progression through activation of Wnt/β-catenin pathway via stabilising FOXM1. Cell Death Dis..

[128] Hao H.X., Xie Y., Zhang Y., Charlat O., Oster E., Avello M., Lei H., Mickanin C., Liu D., Ruffner H. (2012). ZNRF3 promotes Wnt receptor turnover in an R-spondin-sensitive manner. Nature.

[129] Jiang X., Charlat O., Zamponi R., Yang Y., Cong F. (2015). Dishevelled promotes Wnt receptor degradation through recruitment of ZNRF3/RNF43 E3 ubiquitin ligases. Mol. Cell.

[130] Yagyu R., Furukawa Y., Lin Y.M., Shimokawa T., Yamamura T., Nakamura Y. (2004). A novel oncoprotein RNF43 functions in an autocrine manner in colorectal cancer. Int. J. Oncol..

[131] Gonzalez-Sanchez E., Vaquero J., Férnandez-Barrena M.G., Lasarte J.J., Avila M.A., Sarobe P., Reig M., Calvo M., Fabregat I. (2021). The TGF-β Pathway: A Pharmacological Target in Hepatocellular Carcinoma?. Cancers.

[132] Siegel R., Naishadham D., Jemal A. (2013). Cancer statistics, 2013. CA Cancer J. Clin..

[133] Derynck R., Budi E.H. (2019). Specificity, versatility, and control of TGF-β family signaling. Sci. Signal..

[134] Gotzmann J., Fischer A.N., Zojer M., Mikula M., Proell V., Huber H., Jechlinger M., Waerner T., Weith A., Beug H. (2006). A crucial function of PDGF in TGF-beta-mediated cancer progression of hepatocytes. Oncogene.

[135] Kurland J.F., Tansey W.P. (2004). Crashing waves of destruction: The cell cycle and APC(Cdh1) regulation of SCF(Skp2). Cancer Cell.

[136] Hershko A., Ciechanover A. (1998). The ubiquitin system. Annu. Rev. Biochem..

[137] Pavlides S.C., Lecanda J., Daubriac J., Pandya U.M., Gama P., Blank S., Mittal K., Shukla P., Gold L.I. (2016). TGF-β activates APC through Cdh1 binding for Cks1 and Skp2 proteasomal destruction stabilizing p27kip1 for normal endometrial growth. Cell Cycle.

[138] Huang K.T., Pavlides S.C., Lecanda J., Blank S.V., Mittal K.R., Gold L.I. (2012). Estrogen and progesterone regulate p27kip1 levels via the ubiquitin-proteasome system: Pathogenic and therapeutic implications for endometrial cancer. PLoS ONE.

[139] Bai C., Sen P., Hofmann K., Ma L., Goebl M., Harper J.W., Elledge S.J. (1996). SKP1 connects cell cycle regulators to the ubiquitin proteolysis machinery through a novel motif, the F-box. Cell.

[140] Skaar J.R., Pagan J.K., Pagano M. (2013). Mechanisms and function of substrate recruitment by F-box proteins. Nat. Rev. Mol. Cell Biol..

[141] Wang C., Gale M., Keller B.C., Huang H., Brown M.S., Goldstein J.L., Ye J. (2005). Identification of FBL2 as a geranylgeranylated cellular protein required for hepatitis C virus RNA replication. Mol. Cell.

[142] Sandberg J.K., Andersson S.K., Bächle S.M., Nixon D.F., Moll M. (2012). HIV-1 Vpu interference with innate cell-mediated immune mechanisms. Curr. HIV Res..

[143] Toh K.L., Jones C.R., He Y., Eide E.J., Hinz W.A., Virshup D.M., Ptácek L.J., Fu Y.H. (2001). An hPer2 phosphorylation site mutation in familial advanced sleep phase syndrome. Science.

[144] Nelson R.F., Glenn K.A., Miller V.M., Wen H., Paulson H.L. (2006). A novel route for F-box protein-mediated ubiquitination links CHIP to glycoprotein quality control. J. Biol. Chem..

[145] Schröder M., Kaufman R.J. (2005). ER stress and the unfolded protein response. Mutat. Res..

[146] Sun X., Wang T., Guan Z.R., Zhang C., Chen Y., Jin J., Hua D. (2018). FBXO2, a novel marker for metastasis in human gastric cancer. Biochem. Biophys. Res. Commun..

[147] Wei X., Bu J., Mo X., Lv B., Wang X., Hou B. (2018). The prognostic significance of FBXO2 expression in colorectal cancer. Int. J. Clin. Exp. Pathol..

[148] Welcker M., Clurman B.E. (2008). FBW7 ubiquitin ligase: A tumour suppressor at the crossroads of cell division, growth and differentiation. Nat. Rev. Cancer.

[149] Zhou Z., He C., Wang J. (2015). Regulation mechanism of Fbxw7-related signaling pathways (Review). Oncol. Rep..

[150] Gu Z., Inomata K., Ishizawa K., Horii A. (2007). The FBXW7 beta-form is suppressed in human glioma cells. Biochem. Biophys. Res. Commun..

[151] Akhoondi S., Sun D., von der Lehr N., Apostolidou S., Klotz K., Maljukova A., Cepeda D., Fiegl H., Dafou D., Marth C. (2007). FBXW7/hCDC4 is a general tumor suppressor in human cancer. Cancer Res..

[152] Maser R.S., Choudhury B., Campbell P.J., Feng B., Wong K.K., Protopopov A., O’Neil J., Gutierrez A., Ivanova E., Perna I. (2007). Chromosomally unstable mouse tumours have genomic alterations similar to diverse human cancers. Nature.

[153] Urick M.E., Bell D.W. (2018). In vitro effects of FBXW7 mutation in serous endometrial cancer: Increased levels of potentially druggable proteins and sensitivity to SI-2 and dinaciclib. Mol. Carcinog..

[154] Zhang J., Wan L., Dai X., Sun Y., Wei W. (2014). Functional characterization of Anaphase Promoting Complex/Cyclosome (APC/C) E3 ubiquitin ligases in tumorigenesis. Biochim. Biophys. Acta.

[155] Wang Z., Wan L., Zhong J., Inuzuka H., Liu P., Sarkar F.H., Wei W. (2013). Cdc20: A potential novel therapeutic target for cancer treatment. Curr. Pharm. Des..

[156] Clute P., Pines J. (1999). Temporal and spatial control of cyclin B1 destruction in metaphase. Nat. Cell Biol..

[157] Nasmyth K. (2001). Disseminating the genome: Joining, resolving, and separating sister chromatids during mitosis and meiosis. Annu. Rev. Genet..

[158] Jin L., Williamson A., Banerjee S., Philipp I., Rape M. (2008). Mechanism of ubiquitin-chain formation by the human anaphase-promoting complex. Cell.

[159] Di Fiore B., Davey N.E., Hagting A., Izawa D., Mansfeld J., Gibson T.J., Pines J. (2015). The ABBA motif binds APC/C activators and is shared by APC/C substrates and regulators. Dev. Cell.

[160] Wang L., Zhang J., Wan L., Zhou X., Wang Z., Wei W. (2015). Targeting Cdc20 as a novel cancer therapeutic strategy. Pharmacol. Ther..

[161] Bellanger S., Tan C.L., Nei W., He P.P., Thierry F. (2010). The human papillomavirus type 18 E2 protein is a cell cycle-dependent target of the SCFSkp2 ubiquitin ligase. J. Virol..

[162] Bellanger S., Blachon S., Mechali F., Bonne-Andrea C., Thierry F. (2005). High-risk but not low-risk HPV E2 proteins bind to the APC activators Cdh1 and Cdc20 and cause genomic instability. Cell Cycle.

[163] Yu Y., Munger K. (2013). Human papillomavirus type 16 E7 oncoprotein inhibits the anaphase promoting complex/cyclosome activity by dysregulating EMI1 expression in mitosis. Virology.

[164] Chan J.J., Tay Y. (2018). Noncoding RNA:RNA Regulatory Networks in Cancer. Int. J. Mol. Sci..

[165] Ma X., Dang Y., Shao X., Chen X., Wu F., Li Y. (2019). Ubiquitination and Long Non-coding RNAs Regulate Actin Cytoskeleton Regulators in Cancer Progression. Int. J. Mol. Sci..

[166] Vishnubalaji R., Shaath H., Elango R., Alajez N.M. (2020). Noncoding RNAs as potential mediators of resistance to cancer immunotherapy. Semin. Cancer Biol..

[167] Hosseini E.S., Meryet-Figuiere M., Sabzalipoor H., Kashani H.H., Nikzad H., Asemi Z. (2017). Dysregulated expression of long noncoding RNAs in gynecologic cancers. Mol. Cancer.

[168] Qi L., Xu X., Qi X. (2022). The giant E3 ligase HUWE1 is linked to tumorigenesis, spermatogenesis, intellectual disability, and inflammatory diseases. Front. Cell. Infect. Microbiol..

[169] Zammataro L., Lopez S., Bellone S., Pettinella F., Bonazzoli E., Perrone E., Zhao S., Menderes G., Altwerger G., Han C. (2019). Whole-exome sequencing of cervical carcinomas identifies activating ERBB2 and PIK3CA mutations as targets for combination therapy. Proc. Natl. Acad. Sci. USA.

[170] zur Hausen H. (1996). Papillomavirus Infections--a Major Cause of Human Cancers. Biochim. Biophys. Acta.

[171] Scheffner M., Whitaker N.J. (2003). Human Papillomavirus-Induced Carcinogenesis and the Ubiquitin-Proteasome System. Semin. Cancer Biol..

[172] Huh K., Zhou X., Hayakawa H., Cho J.Y., Libermann T.A., Jin J., Harper J.W., Munger K. (2007). Human Papillomavirus Type 16 E7 Oncoprotein Associates with the Cullin 2 Ubiquitin Ligase Complex, Which Contributes to Degradation of the Retinoblastoma Tumor Suppressor. J. Virol..

[173] Wang Y., Zheng Z., Zhang J., Wang Y., Kong R., Liu J., Zhang Y., Deng H., Du X., Ke Y. (2015). A Novel Retinoblastoma Protein (Rb) E3 Ubiquitin Ligase (Nrbe3) Promotes Rb Degradation and Is Transcriptionally Regulated by E2f1 Transcription Factor. J. Biol. Chem..

[174] Wu M.X. (2003). Roles of the stress-induced gene IEX-1 in regulation of cell death and oncogenesis. Apoptosis.

[175] Arlt A., Schäfer H. (2011). Role of the immediate early response 3 (IER3) gene in cellular stress response, inflammation and tumorigenesis. Eur. J. Cell Biol..

[176] Arlt A., Grobe O., Sieke A., Kruse M.L., Fölsch U.R., Schmidt W.E., Schäfer H. (2001). Expression of the NF-kappa B target gene IEX-1 (p22/PRG1) does not prevent cell death but instead triggers apoptosis in Hela cells. Oncogene.

[177] Schilling D., Pittelkow M.R., Kumar R. (2001). IEX-1, an immediate early gene, increases the rate of apoptosis in keratinocytes. Oncogene.

[178] Li H.Y., Kotaka M., Kostin S., Lee S.M., Kok L.D., Chan K.K., Tsui S.K., Schaper J., Zimmermann R., Lee C.Y. (2001). Translocation of a human focal adhesion LIM-only protein, FHL2, during myofibrillogenesis and identification of LIM2 as the principal determinants of FHL2 focal adhesion localization. Cell Motil. Cytoskelet..

[179] Johannessen M., Møller S., Hansen T., Moens U., Van Ghelue M. (2006). The multifunctional roles of the four-and-a-half-LIM only protein FHL2. Cell. Mol. Life Sci..

[180] Hayashi H., Nakagami H., Takami Y., Koriyama H., Mori M., Tamai K., Sun J., Nagao K., Morishita R., Kaneda Y. (2009). FHL-2 suppresses VEGF-induced phosphatidylinositol 3-kinase/Akt activation via interaction with sphingosine kinase-1. Arterioscler. Thromb. Vasc. Biol..

[181] Kadrmas J.L., Beckerle M.C. (2004). The LIM domain: From the cytoskeleton to the nucleus. Nat. Rev. Mol. Cell Biol..

[182] Zhao Y., Hu X., Wei L., Song D., Wang J., You L., Saiyin H., Li Z., Yu W., Yu L. (2018). PARP10 suppresses tumor metastasis through regulation of Aurora A activity. Oncogene.

[183] Yu M., Schreek S., Cerni C., Schamberger C., Lesniewicz K., Poreba E., Vervoorts J., Walsemann G., Grötzinger J., Kremmer E. (2005). PARP-10, a novel Myc-interacting protein with poly(ADP-ribose) polymerase activity, inhibits transformation. Oncogene.

[184] Ding B., Liang H., Gao M., Li Z., Xu C., Fan S., Chang N. (2017). Forkhead Box A2 (FOXA2) Inhibits Invasion and Tumorigenesis in Glioma Cells. Oncol. Res..

[185] Tang Y., Shu G., Yuan X., Jing N., Song J. (2011). FOXA2 functions as a suppressor of tumor metastasis by inhibition of epithelial-to-mesenchymal transition in human lung cancers. Cell Res..

[186] Kuratomi G., Komuro A., Goto K., Shinozaki M., Miyazawa K., Miyazono K., Imamura T. (2005). NEDD4-2 (neural precursor cell expressed, developmentally down-regulated 4-2) negatively regulates TGF-beta (transforming growth factor-beta) signalling by inducing ubiquitin-mediated degradation of Smad2 and TGF-beta type I receptor. Biochem. J..

[187] Hellwinkel O.J., Asong L.E., Rogmann J.P., Sültmann H., Wagner C., Schlomm T., Eichelberg C. (2011). Transcription alterations of members of the ubiquitin-proteasome network in prostate carcinoma. Prostate Cancer Prostatic Dis..

[188] Orlowski R.Z., Stinchcombe T.E., Mitchell B.S., Shea T.C., Baldwin A.S., Stahl S., Adams J., Esseltine D.L., Elliott P.J., Pien C.S. (2002). Phase I trial of the proteasome inhibitor PS-341 in patients with refractory hematologic malignancies. J. Clin. Oncol..

[189] Kawabata S., Gills J.J., Mercado-Matos J.R., Lopiccolo J., Wilson W., Hollander M.C., Dennis P.A. (2012). Synergistic effects of nelfinavir and bortezomib on proteotoxic death of NSCLC and multiple myeloma cells. Cell Death Dis..

[190] Shen Y., Lu L., Xu J., Meng W., Qing Y., Liu Y., Zhang B., Hu H. (2013). Bortezomib induces apoptosis of endometrial cancer cells through microRNA-17-5p by targeting p21. Cell Biol. Int..

[191] Sun N.K., Huang S.L., Chang T.C., Chao C.C. (2013). Sorafenib induces endometrial carcinoma apoptosis by inhibiting Elk-1-dependent Mcl-1 transcription and inducing Akt/GSK3β-dependent protein degradation. J. Cell. Biochem..

[192] Piccinini M., Rinaudo M.T., Chiapello N., Ricotti E., Baldovino S., Mostert M., Tovo P.A. (2002). The human 26S proteasome is a target of antiretroviral agents. Aids.

[193] Lv Z., Chu Y., Wang Y. (2015). HIV protease inhibitors: A review of molecular selectivity and toxicity. HIV AIDS.

[194] Gaedicke S., Firat-Geier E., Constantiniu O., Lucchiari-Hartz M., Freudenberg M., Galanos C., Niedermann G. (2002). Antitumor effect of the human immunodeficiency virus protease inhibitor ritonavir: Induction of tumor-cell apoptosis associated with perturbation of proteasomal proteolysis. Cancer Res..

[195] Chow W.A., Jiang C., Guan M. (2009). Anti-HIV drugs for cancer therapeutics: Back to the future?. Lancet Oncol..

[196] Toschi E., Sgadari C., Malavasi L., Bacigalupo I., Chiozzini C., Carlei D., Compagnoni D., Bellino S., Bugarini R., Falchi M. (2011). Human immunodeficiency virus protease inhibitors reduce the growth of human tumors via a proteasome-independent block of angiogenesis and matrix metalloproteinases. Int. J. Cancer.

[197] Hampson L., Kitchener H.C., Hampson I.N. (2006). Specific HIV protease inhibitors inhibit the ability of HPV16 E6 to degrade p53 and selectively kill E6-dependent cervical carcinoma cells in vitro. Antivir. Ther..

[198] Dolma S., Lessnick S.L., Hahn W.C., Stockwell B.R. (2003). Identification of genotype-selective antitumor agents using synthetic lethal chemical screening in engineered human tumor cells. Cancer Cell.

[199] Berkson R.G., Hollick J.J., Westwood N.J., Woods J.A., Lane D.P., Lain S. (2005). Pilot screening programme for small molecule activators of p53. Int. J. Cancer.

[200] Thangasamy T., Sittadjody S., Lanza-Jacoby S., Wachsberger P.R., Limesand K.H., Burd R. (2007). Quercetin selectively inhibits bioreduction and enhances apoptosis in melanoma cells that overexpress tyrosinase. Nutr. Cancer.

[201] Udomwan P., Pientong C., Tongchai P., Burassakarn A., Sunthamala N., Roytrakul S., Suebsasana S., Ekalaksananan T. (2021). Proteomics Analysis of Andrographolide-Induced Apoptosis via the Regulation of Tumor Suppressor p53 Proteolysis in Cervical Cancer-Derived Human Papillomavirus 16-Positive Cell Lines. Int. J. Mol. Sci..

[202] Mthembu N.N., Motadi L.R. (2014). Apoptotic potential role of Agave palmeri and Tulbaghia violacea extracts in cervical cancer cells. Mol. Biol. Rep..

[203] Wang C., Zhang Y., Wu Y., Xing D. (2021). Developments of Crbn-Based Protacs as Potential Therapeutic Agents. Eur. J. Med. Chem..

[204] Zhao C.Y., Szekely L., Bao W., Selivanova G. (2010). Rescue of p53 function by small-molecule RITA in cervical carcinoma by blocking E6-mediated degradation. Cancer Res..

[205] Liu L., Yu Z.Y., Yu T.T., Cui S.H., Yang L., Chang H., Qu Y.H., Lv X.F., Zhang X.A., Ren C.C. (2020). A Slug-dependent mechanism is responsible for tumor suppression of p53-stabilizing compound CP-31398 in p53-mutated endometrial carcinoma. J. Cell. Physiol..

[206] Pavlides S.C., Huang K.T., Reid D.A., Wu L., Blank S.V., Mittal K., Guo L., Rothenberg E., Rueda B., Cardozo T. (2013). Inhibitors of SCF-Skp2/Cks1 E3 ligase block estrogen-induced growth stimulation and degradation of nuclear p27kip1: Therapeutic potential for endometrial cancer. Endocrinology.

[207] Talis A.L., Huibregtse J.M., Howley P.M. (1998). The role of E6AP in the regulation of p53 protein levels in human papillomavirus (HPV)-positive and HPV-negative cells. J. Biol. Chem..

[208] Clemente-Soto A.F., Salas-Vidal E., Milan-Pacheco C., Sánchez-Carranza J.N., Peralta-Zaragoza O., González-Maya L. (2019). Quercetin induces G2 phase arrest and apoptosis with the activation of p53 in an E6 expression-independent manner in HPV-positive human cervical cancer-derived cells. Mol. Med. Rep..

[209] Srivastava S., Somasagara R.R., Hegde M., Nishana M., Tadi S.K., Srivastava M., Choudhary B., Raghavan S.C. (2016). Quercetin, a Natural Flavonoid Interacts with DNA, Arrests Cell Cycle and Causes Tumor Regression by Activating Mitochondrial Pathway of Apoptosis. Sci. Rep..

[210] Brooks C.L., Gu W. (2006). p53 ubiquitination: Mdm2 and beyond. Mol. Cell.

[211] Wade M., Wahl G.M. (2009). Targeting Mdm2 and Mdmx in cancer therapy: Better living through medicinal chemistry?. Mol. Cancer Res..

[212] Sekine K., Takubo K., Kikuchi R., Nishimoto M., Kitagawa M., Abe F., Nishikawa K., Tsuruo T., Naito M. (2008). Small molecules destabilize cIAP1 by activating auto-ubiquitylation. J. Biol. Chem..

[213] Zhang X., Linder S., Bazzaro M. (2020). Drug Development Targeting the Ubiquitin-Proteasome System (UPS) for the Treatment of Human Cancers. Cancers.

[214] Luo Z., Yu G., Lee H.W., Li L., Wang L., Yang D., Pan Y., Ding C., Qian J., Wu L. (2012). The Nedd8-activating enzyme inhibitor MLN4924 induces autophagy and apoptosis to suppress liver cancer cell growth. Cancer Res..

[215] Liu H., Bei Q., Luo X. (2021). MLN4924 inhibits cell proliferation by targeting the activated neddylation pathway in endometrial carcinoma. J. Int. Med. Res.

[216] Ostertag M.S., Hutwelker W., Plettenburg O., Sattler M., Popowicz G.M. (2019). Structural Insights into BET Client Recognition of Endometrial and Prostate Cancer-Associated SPOP Mutants. J. Mol. Biol..

[217] Crews C.M. (2010). Targeting the undruggable proteome: The small molecules of my dreams. Chem. Biol..

[218] Lai A.C., Crews C.M. (2017). Induced protein degradation: An emerging drug discovery paradigm. Nat. Rev. Drug Discov..

[219] Toure M., Crews C.M. (2016). Small-Molecule PROTACS: New Approaches to Protein Degradation. Angew. Chem. Int. Ed. Engl..

[220] Benowitz A.B., Jones K.L., Harling J.D. (2021). The therapeutic potential of PROTACs. Expert Opin. Ther. Pat..

[221] Filippakopoulos P., Qi J., Picaud S., Shen Y., Smith W.B., Fedorov O., Morse E.M., Keates T., Hickman T.T., Felletar I. (2010). Selective inhibition of BET bromodomains. Nature.

[222] Zengerle M., Chan K.H., Ciulli A. (2015). Selective Small Molecule Induced Degradation of the BET Bromodomain Protein BRD4. ACS Chem. Biol..

[223] Ward C.C., Kleinman J.I., Brittain S.M., Lee P.S., Chung C.Y.S., Kim K., Petri Y., Thomas J.R., Tallarico J.A., McKenna J.M. (2019). Covalent Ligand Screening Uncovers a RNF4 E3 Ligase Recruiter for Targeted Protein Degradation Applications. ACS Chem. Biol..

[224] Ishida T., Ciulli A. (2021). E3 Ligase Ligands for PROTACs: How They Were Found and How to Discover New Ones. SLAS Discov..

[225] Paiva S.L., Crews C.M. (2019). Targeted protein degradation: Elements of PROTAC design. Curr. Opin. Chem. Biol..

[226] Li X., Song Y. (2020). Proteolysis-targeting chimera (PROTAC) for targeted protein degradation and cancer therapy. J. Hematol. Oncol..

[227] Xi M., Chen Y., Yang H., Xu H., Du K., Wu C., Xu Y., Deng L., Luo X., Yu L. (2019). Small molecule PROTACs in targeted therapy: An emerging strategy to induce protein degradation. Eur. J. Med. Chem..

[228] Zou Y., Ma D., Wang Y. (2019). The PROTAC technology in drug development. Cell Biochem. Funct..

